# Sequential release of bioactive factors from functionalized metal-organic framework hydrogel enhances interfacial osseointegration of 3D-printed titanium alloy porous scaffolds

**DOI:** 10.7150/thno.120711

**Published:** 2026-01-01

**Authors:** Zhenjia Che, Xiao Sheng, Yanglin Wu, Binghao Lin, Kaihang Song, Qiyun Chen, Aopan Chen, Lingxuan Deng, Jing Chen, Ming Cai

**Affiliations:** 1 Department of Orthopaedics, Shanghai Tenth People's Hospital, School of Medicine, Tongji University, No.301 Middle Yanchang Road, Shanghai 200072, P. R. China.; 2 Huzhou Central Hospital, Fifth School of Clinical Medicine of Zhejiang Chinese Medical University, Wuxing, Huzhou, Zhejiang 313000, P. R. China.

**Keywords:** sequential release, 3D-printed porous titanium scaffold, osteointegration, osteoporosis, intelligent hydrogel

## Abstract

**Rationale:** Repairing bone defects in osteoporotic patients presents a significant clinical challenge due to inadequate osseointegration, persistent inflammation, and elevated oxidative stress. To overcome these barriers, this study proposes the development of a functionalized 3D-printed titanium alloy porous scaffold capable of sequentially releasing therapeutic agents to modulate the immune environment and enhance bone regeneration.

**Methods:** A thermosensitive collagen hydrogel was integrated with a zeolitic imidazolate framework (ZIF-8) to construct a dual-release platform capable of delivering the immunomodulator 4-octyl itaconate (4-OI) and the osteogenic factor bone morphogenetic protein-9 (BMP-9) in a temporally controlled manner. The hydrogel facilitated early-phase release of 4-OI to inhibit M1 macrophage polarization and mitigate oxidative stress, while ZIF-8 enabled sustained BMP-9 release to induce osteogenic differentiation of bone marrow mesenchymal stem cells (BMSCs). Comprehensive in vitro assays and an osteoporotic rat model were employed to evaluate the scaffold's immunomodulatory properties, osteogenic capacity, and osseointegration performance.

**Results:** The scaffold inhibited pro-inflammatory cytokine expression, attenuated osteoclast activity, and enhanced osteogenic marker levels in vitro. In vivo analysis revealed enhanced bone-implant interface integration and significantly accelerated bone regeneration in osteoporotic defects. Transcriptome analysis revealed suppression of NF-κB and TGF-β signaling, confirming the scaffold's combined immunomodulatory and osteoinductive effects.

**Conclusions:** This ZIF-functionalized hydrogel scaffold with sequential release capability offers a potential strategy for clinical translation in osteoporotic bone defect repair. By orchestrating local immune modulation and promoting sustained osteogenesis, the system offers a clinically relevant approach to enhance osseointegration and facilitate long-term bone repair in osteoporotic conditions.

## Introduction

Osteoporosis is a systemic condition characterized by disruptions in bone metabolism, primarily resulting in diminished bone formation and heightened bone resorption, leading to increased fragility and a higher susceptibility to fractures. Currently, bone defects caused by various reasons usually require surgical intervention 1-3. Factors such as trauma, tumor resection, and infections often induce structural damage to bones in osteoporotic patients, giving rise to bone defects characterized by compromised bone quality, delayed healing, and elevated recurrence rates 4. This highlights the urgent need for tailored implant materials capable of effectively addressing bone defects, promoting osteogenesis in osteoporosis-related lesions, and supporting optimal tissue repair and integration 5-7.

Titanium and its alloys are widely used in orthopedic applications due to their excellent biocompatibility and mechanical properties. However, their biological inertness limits their ability to stimulate bone regeneration, as they lack adequate bioactivity. Bone regeneration progresses through three main stages: immune regulation, cellular proliferation with tissue formation, and subsequent remodeling and maturation 8, 9. Inadequate regulation of these phases can result in poor implant-bone integration 10, 11. To enhance the bioactivity of titanium implants, several physical and chemical surface modifications have been explored, including topographical alterations, osteogenic factor immobilization, and doping with functional ions. While these strategies have improved osteointegration to some extent 12, 13, their clinical effectiveness in osteoporosis remains limited, largely owing to abnormal immune responses elicited by foreign materials and the unfavorable microenvironment with elevated reactive oxygen species (ROS). Thus, focusing solely on the osteogenic properties of the material, without addressing the local immune microenvironment, is insufficient for optimal bone regeneration. An ideal implant material must possess both osteoinductive and immunoregulatory properties, working synergistically to align with bone regeneration processes and foster effective bone-implant integration 14.

To maintain immune regulation and improve metal-bone integration, various approaches have been explored. For instance, Wang et al. 15 developed a dual-functional surface coating for titanium screws. Mussel-derived peptides with clickable groups were synthesized and combined with azide-modified bone morphogenetic protein-2 (BMP-2) peptides. These mussel-derived peptides were immobilized on the titanium screw surface *via* metal-catechol coordination, followed by zinc ion (Zn^2+^) loading and BMP-2 peptide attachment. This surface modification induced a shift in macrophages from the pro-inflammatory M1 state toward the anti-inflammatory M2 phenotype, which optimized the local immune milieu and supported osteogenic differentiation of bone marrow mesenchymal stem cells. This approach significantly improved bone-implant integration and facilitated new bone formation. Despite these advancements in immune modulation and bone regeneration, the lack of temporal control over the release of Zn^2+^ and BMP-2 peptides limits the osteogenic potential. Thus, developing scaffolds capable of sequentially releasing multiple bioactive molecules or growth factors in a controlled manner is essential to achieve optimal bone integration outcomes.

Recently, hydrogels have gained wide interest owing to their unique characteristics. These materials, formed by the crosslinking of hydrophilic polymers, can absorb substantial amounts of water and exhibit mechanical characteristics similar to human tissues, making them ideal candidates for tissue engineering applications 16-18. Hydrogels, whether derived from natural or synthetic polymers, are widely utilized for the delivery of bioactive molecules. By chelating these molecules, hydrogels prevent their rapid enzymatic degradation or inactivation while modulating their release rates to sustain prolonged *in vivo* activity and minimize nonspecific diffusion. Thermoresponsive collagen hydrogel (Col), with its excellent biocompatibility, biodegradability, viscoelasticity, viscosity, and permeability, offers a unique advantage. Leveraging its thermoresponsive nature, Col forms a homogeneous solution with bioactive molecules at lower temperatures, transitioning swiftly into a gel state at body temperature (37 °C). Furthermore, 4-Octyl itaconate (4-OI), an esterified form of itaconate, exhibits anti-inflammatory and antioxidant activities by suppressing succinate dehydrogenase (SDH) and activating Nrf2, making it a potential mitochondrial-targeted immunoregulatory molecule 19. It was physically blended with thermosensitive Col as a release system. As the Col gradually degrades, it releases 4-OI locally in a controlled, sustained, and slow manner.

Metal-organic frameworks (MOFs) have found extensive applications in various domains, including catalysis, gas adsorption/separation/storage, nonlinear optics, sensing, and biomedicine, owing to their ease of synthesis, functionalization, tunable pore sizes, structural diversity, high surface area, strong drug-loading capacity, biocompatibility, and degradability 20-22. Zeolitic imidazolate framework-8 (ZIF-8), a prominent subclass of MOFs, exhibit excellent biocompatibility and serve as protective carriers for biomacromolecules, safeguarding them from inactivation caused by high temperatures, organic solvents, and protein hydrolysis. Constructed by coordinating Zn^2+^ with 2-methylimidazole (2-MIM), ZIF-8 demonstrates low cytotoxicity, high drug-loading efficiency, and pH-responsive degradation 23. With its unique porous structure, high loading capacity, pH sensitivity, antibacterial properties, and exceptional thermal and chemical stability, ZIF-8 holds great promise for protein and growth factor delivery systems. Niu et al. 24 developed nanoparticles by loading BMP-2 into ZIF-8, which were then incorporated into a sodium alginate/hydroxyapatite/polyvinyl alcohol hydrogel containing PDGF-BB. This strategy facilitated the sequential release of angiogenic and osteogenic factors during bone repair, significantly enhancing neovascularization and bone tissue regeneration.

Building upon this research, a 3D-printed titanium alloy micro-porous scaffold capable of sequentially releasing immunomodulatory agents and osteogenic growth factors was developed. This scaffold not only stabilizes bioactive substances but also optimizes the release sequence of multiple active factors. Thermosensitive Col serves as the primary sustained-release platform, with 4-OI incorporated and gradually released in a controlled manner as the hydrogel degrades during the early stages of implantation. This controlled release modulates immune responses, alleviates local inflammation, and scavenges ROS. As a secondary release platform, BMP-9, an osteogenic factor, is encapsulated within ZIF-8, utilizing its pH sensitivity for sustained release. BMP-9 is initially released into the hydrogel matrix, then diffuses into the surrounding tissue as the hydrogel degrades, effectively maintaining the active concentration of growth factors within the therapeutic region and supporting continuous bone integration. Compared to traditional titanium implants, the sequential release scaffold developed in this study provides significant advantages in regulating the microenvironment across various pathological stages, minimizing drug side effects, and sustaining local therapeutic efficacy, providing a potential approach for effective osteoporotic bone defect repair with clinical applicability.

## Results and Discussion

### Synthesis and Characterization of ZIF-8@BMP-9 Nanoparticles

**Scheme 1:**
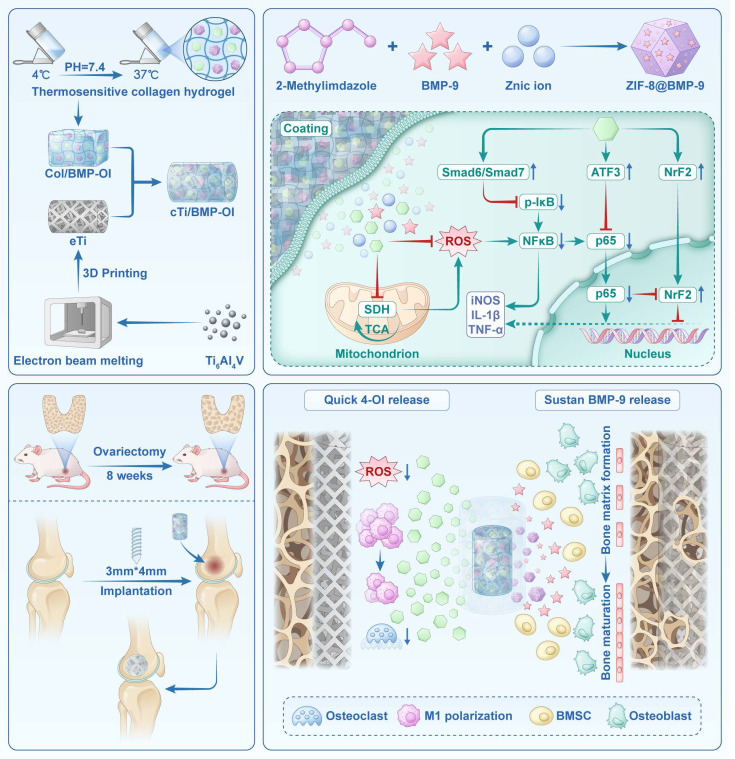
Schematic illustration of the synthesis and application of cTi/BMP-OI, designed to enhance the integration of the metal-bone interface under osteoporotic conditions.

**Figure 1:**
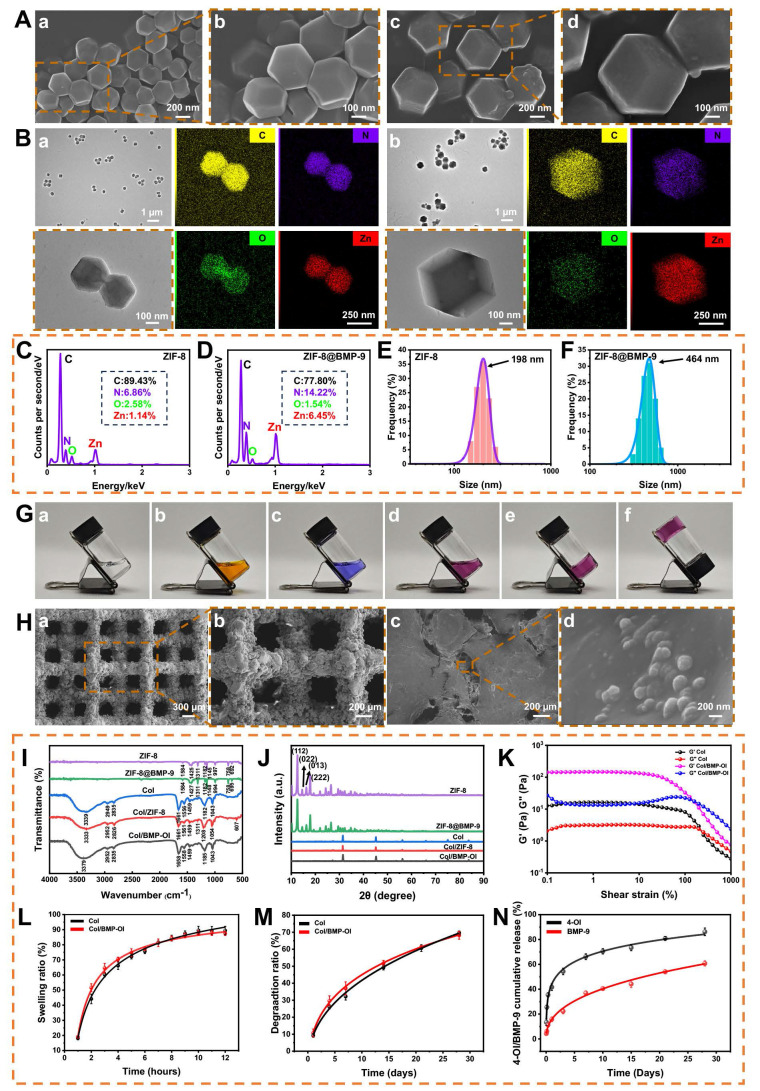
Characterization of cTi/BMP-OI. (A) SEM images: (a, b) ZIF-8; (c, d) ZIF@BMP-9. (B) TEM micrographs with corresponding EDS spectra: (a) ZIF-8; (b) ZIF@BMP-9. (C) and (D) Elemental distribution maps of ZIF-8 and ZIF@BMP-9. (E) and (F) Size distribution of ZIF-8 and ZIF-8@BMP-9 from the measurement of TEM images. (G) Thermoresponsive collagen hydrogels. (a) type I collagen; (b) MEM culture medium; (c) buffer solution; (d) Temperature-sensitive collagen hydrogel in the 4 °C; (e) and (f) Temperature-sensitive collagen hydrogel in the 37 °C. (H) SEM, (a) and (b): eTi; (c) and (d): cTi/BMP-OI. (I) FTIR of ZIF-8, ZIF@BMP-9, Col, Col/ZIF-8 and Col/BMP-OI. (J) XRD of ZIF-8, ZIF@BMP-9, Col, Col/ZIF-8 and Col/BMP-OI. (K) G′ and G″ of Col and Col/BMP-OI varied with shear strain. (L) Swelling ratio curves of Col and Col/BMP-OI. (M) Degradation curves of Col and Col/BMP-OI. (N) 4-OI and BMP-9 release from cTi/BMP-OI over 28 days in vitro.

ZIF-8 and ZIF-8@BMP-9 nanoparticles were synthesized using a one-pot method as described in the literature 25, with the specific preparation process outlined in **Scheme 1**. Visual inspection revealed that both ZIF-8 and ZIF-8@BMP-9 exhibited a milky white dispersion (**Figure S1**). Scanning electron microscopy (SEM) and transmission electron microscopy (TEM) images (**Figure 1A-B**) confirmed that both nanoparticles displayed a regular dodecahedral morphology. Notably, the encapsulation of BMP-9 did not significantly alter the structural morphology, although ZIF-8@BMP-9 particles were slightly larger than ZIF-8. Both materials were synthesized through coordination between Zn^2+^ and 2-MIM to form the MOF. Energy-dispersive X-ray spectroscopy (EDS) elemental mapping revealed uniform distribution of Zn, C, O, and N elements throughout the samples (**Figure 1B**). Additionally, nitrogen content in ZIF-8 and ZIF-8@BMP-9 was measured at 6.68% and 14.22%, respectively, indicating a significant increase in nitrogen levels following BMP-9 encapsulation (**Figure 1C-D**). Dynamic light scattering (DLS) revealed the hydrodynamic diameters of ZIF-8 and ZIF-8@BMP-9 were 198 ± 25 nm and 460 ± 54 nm, respectively (**Figure 1E-F**). An enzyme-linked immunosorbent assay (ELISA) was performed to determine the residual BMP-9 in the supernatant after encapsulation, thereby evaluating the encapsulation efficiency. The analysis indicated that approximately 95.69% of BMP-9 was successfully encapsulated.

### Development of Thermoresponsive Collagen Hydrogel for Sequential Drug Delivery

The thermoresponsive behavior of the hydrogel was assessed using the small bottle inversion method 26. At 37 °C, type I collagen (**Figure 1G(a)**), MEM culture medium (**Figure 1G(b)**), and buffer solution (**Figure 1G(c)**) remained in liquid form. However, as shown in **Figure 1G(d)**, the thermoresponsive Col displayed free-flowing properties at 4 °C, transitioning rapidly into a gel state at 37 °C within 10 min, demonstrating its thermal-responsive gelation characteristics. At 37 °C, collagen molecular chains intertwine and crosslink through hydrogen bonding and hydrophobic interactions, forming a three-dimensional network structure that enhances the hydrogel's gel strength and stability **(Figure 1G(e-f)**).

### Fabrication and Structural Characterization of Composite Titanium Scaffold

In this study, a porous titanium alloy implant was successfully fabricated using 3D printing technology (**Figure S2**). Low-magnification SEM images showed that the struts of the empty titanium alloy scaffold (eTi) formed a regular "micropore" structure with 90° right-angle interconnections (**Figure 1H(a)**). High-magnification SEM images revealed that the eTi scaffold surface was smooth and flat, free of impurities (**Figure 1H(b)**). The thermoresponsive Col, ZIF-8@BMP-9, and 4-OI were physically blended at 4 °C and combined with the titanium alloy scaffold to form the composite scaffold. At low magnification, the hydrogel effectively filled the scaffold's pores and uniformly covered its surface (**Figure 1H(c)**). Further high-magnification analysis confirmed that ZIF-8@BMP-9 nanoparticles were successfully embedded in the hydrogel and adhered to the titanium alloy surface, verifying the successful construction of the composite material (**Figure 1H(d)**). EDS characterization of eTi, titanium alloy scaffold loaded with thermoresponsive Col (cTi), and titanium alloy scaffold with sequential release of 4-OI and BMP-9 (cTi/BMP-OI) scaffolds (**Figure S3-5**) confirmed the presence of significant Zn element expression in the cTi/BMP-OI sample, corresponding to the characteristic ZIF-8 element, which serves as the carrier for BMP-9. This provided preliminary evidence for the effective loading of functional components into the composite material.

Fourier-transform infrared spectroscopy (FTIR) analysis was performed to explore the chemical structures of ZIF-8, ZIF-8@BMP-9, Col, Col/ZIF-8, and Col/BMP-OI (**Figure 1I**). Distinct absorption peaks were observed across all groups, confirming their unique chemical bonding characteristics. In the FTIR spectrum of ZIF-8, clear vibration peaks corresponding to the Zn-N coordination bond, as well as C=N and C-N bonds in the imidazole ring, were identified, confirming the successful synthesis of ZIF-8. For ZIF-8@BMP-9, the intensity of the FTIR absorption peaks notably increased, especially at 1148 cm^-1^, where a significant peak enhancement was observed. Despite this, the key vibration peaks of the ZIF-8 framework remained largely unchanged, suggesting the preservation of the ZIF-8 crystal structure. This implies that BMP-9 was integrated into the ZIF-8 framework rather than simply adsorbed on its surface. Furthermore, the observed peak enhancements and minor shifts further supported the successful formation of the ZIF-8@BMP-9 composite, indicating efficient BMP-9 loading without disrupting the MOF structure.

In the FTIR spectrum of the Col, characteristic absorption peaks for amide I (C=O stretching vibration), amide II (N-H bending vibration), and functional groups such as C-H and C-O were detected, confirming that the Col maintained its natural secondary structure. Upon incorporating ZIF-8 or ZIF-8@BMP-9, changes in the infrared absorption spectrum of the collagen were observed. Specifically, the intensity of the N-H stretching vibration peak decreased, suggesting potential interactions between Zn^2+^ in ZIF-8 and the amide groups of collagen molecules, leading to the formation of coordination bonds. However, absorption peaks related to C-H bonds and other hydrophobic side chains remained largely unchanged, indicating that the collagen backbone structure remained intact. Additionally, no significant shifts were observed in the C-O vibration peaks, suggesting that the hydrogel's chemical environment was not disrupted. These findings suggest that ZIF-8 and BMP-9 nanoparticles reinforce the hydrogel network via intermolecular interactions, thereby improving its mechanical strength and overall functionality.

As shown in **Figure 1J**, the X-ray diffraction (XRD) pattern of ZIF-8 displays several sharp diffraction peaks corresponding to its characteristic crystal faces (112), (022), (013), and (222), confirming its high crystallinity and well-defined crystalline structure. After BMP-9 encapsulation, the ZIF-8@BMP-9 sample maintained the primary diffraction peaks of ZIF-8, with no significant shifts in peak positions or the appearance of new peaks. This suggests that BMP-9 incorporation did not disrupt the ZIF-8 crystal structure, indicating that BMP-9 is likely encapsulated within the ZIF-8 pores without altering its crystalline form. In contrast, the XRD pattern of Col exhibits a relatively flat diffraction background, characteristic of an amorphous structure. Upon integrating ZIF-8 or ZIF-8@BMP-9 into the collagen matrix, the characteristic diffraction peaks of ZIF-8 became clearly visible, confirming the successful incorporation of ZIF-8 into the collagen structure. The absence of new diffraction peaks indicates that the interaction between ZIF-8 and collagen is primarily due to physical encapsulation rather than the formation of a new crystalline structure or covalent bonds. FTIR and XRD analyses validate the successful encapsulation of BMP-9 within the ZIF-8 framework. The subsequent integration of this framework into the thermoresponsive Col ensures efficient BMP-9 loading. This mild, non-destructive encapsulation method preserves the integrity of the ZIF-8 crystalline structure while enabling controlled release of BMP-9, presenting considerable promise for biomedical applications.

### Rheological Behavior and Mechanical Performance of Composite Hydrogel

As shown in **Figure 1K and S6A-B**, the rheological properties of the temperature-sensitive Col and its composite form incorporating ZIF-8@BMP-9 were evaluated. The results indicate that, under low shear strain, both hydrogels exhibited stable storage modulus (G') and loss modulus (G"), with G' consistently higher than G", suggesting that both hydrogels remained in a stable, elastic solid state. As shear strain increased, both G' and G" gradually decreased, signaling hydrogel structural breakdown and a clear rheological response. As presented in **Figure S6A**, the Col demonstrated good elasticity at low strain levels, but the moduli rapidly decreased at higher strains, indicating a loss of structural stability. In contrast, the composite hydrogel containing ZIF-8@BMP-9 maintained superior modulus retention across the entire strain range, with only a slight decline in modulus, indicating enhanced network stability and resistance to deformation (**Figure S6B**). These results demonstrate that the addition of ZIF-8@BMP-9 significantly improved the mechanical properties of the Col, enhancing its ability to maintain structural integrity when implanted in bone defect areas. This suggests that the composite hydrogel is more resilient and is therefore an ideal material for biomedical applications, particularly in bone tissue engineering.

As illustrated in **Figure S6A-B**, the rheological behavior of both the pure Col and its composite with ZIF-8@BMP-9 was assessed in terms of shear force and viscosity. As shown in **Figure S6A**, the pure Col exhibited high and stable viscosity at low shear rates, indicating structural stability and good shear resistance. As the shear rate increased, viscosity gradually decreased while shear force increased, suggesting the hydrogel began to flow and became more deformable under higher shear stress. In contrast, the composite hydrogel with ZIF-8@BMP-9 exhibited higher initial viscosity at low shear rates, indicating that the incorporation of ZIF-8@BMP-9 enhanced the hydrogel's structural stability and shear resistance (**Figure S6B**). Furthermore, under high shear rates, the composite hydrogel's viscosity decreased to a lesser extent, and the change in shear force was more gradual, demonstrating superior rheological stability and flowability compared to the pure Col. Overall, the incorporation of ZIF-8@BMP-9 significantly improved the hydrogel's ability to maintain its structure under dynamic conditions, reducing the shear force required for flow, and enhancing its processing performance and resistance to deformation. These improvements make the composite hydrogel more adaptable to complex physiological environments, further bolstering its suitability for biomedical applications, such as bone tissue engineering.

### Swelling Behavior, Biodegradability, and Sequential Release Performance

In this study, the Col reached swelling equilibrium within 10 h, with a final swelling ratio of 110% (**Figure 1L**). The incorporation of ZIF-8@BMP-9 resulted in a swelling ratio that remained close to 110%, with no significant statistical difference compared to the non-composite group. However, the composite hydrogel exhibited a slightly reduced swelling rate, likely due to the increased crosslinking density induced by ZIF-8@BMP-9. This enhanced crosslinking density likely restricted the hydrogel's water absorption capacity to some extent. Despite this, the composite hydrogel maintained its ability to facilitate nutrient and gas exchange, indicating that the increase in crosslinking did not significantly compromise the hydrogel's biological compatibility. These results suggest that the incorporation of ZIF-8@BMP-9 does not impair the hydrogel's overall biocompatibility while helping to preserve its functional integrity, allowing it to continue supporting essential biological interactions. This characteristic is critical for its applications in tissue engineering and regenerative medicine.

The degradation behavior of the thermosensitive Col demonstrated distinct temporal and pH value variations. Initially, as the hydrogel absorbed water and swelled, both its volume and weight increased. However, with continued degradation, both the volume and weight of the hydrogel gradually decreased, and the degradation time was more than 28 days (**Figure 1M**). At different pH values (6.5, 7.4, and 8.5), thermosensitive Col and functionalized thermosensitive Col exhibited obvious pH-dependent degradation behaviors (**Figure S7A-B**). Under pH 7.4 conditions, the triple-helix structure of collagen was well maintained, and the hydrogels showed the highest structural stability with the slowest degradation rates, approximately 69.61 ± 1.10% and 68.21 ± 2.32%, serving as the control condition simulating the in vivo environment. The degradation rate was fastest at pH 8.5, reaching approximately 77.04 ± 3.06% at 28 days, which may be related to the destruction of hydrogen bonds and the enhancement of hydrolysis reactions in an alkaline environment. This led to a rapid loosening of the collagen network structure, resulting in a significantly accelerated degradation rate. At pH 6.5, the degradation rates were approximately 73.53 ± 0.56% and 76.47 ± 0.94%, slightly faster than under neutral conditions, which may be associated with conformational relaxation caused by changes in the charge state of collagen molecules. This reduced the structural stability of the hydrogels and moderately increased the degradation rate. This process demonstrates the hydrogel's excellent swelling properties and biodegradability, making it a promising candidate for various tissue engineering applications. The degradation rate of the hydrogel is influenced by factors such as crosslinking degree, solvent type, and environmental conditions, all contributing to its stability and degradation behavior. By adjusting the crosslinking density and composition, the degradation rate can be precisely controlled to meet the specific clinical needs of different applications.

As a scaffold material, the Col offers significant advantages in tissue engineering, particularly in its ability to control the release of bioactive substances. The sequential release of these substances not only promotes the integration of metal and bone interfaces but also facilitates bone repair. During the early release phase, the large surface area of the hydrogel in contact with simulated body fluid results in a relatively rapid degradation rate, leading to a burst release of 4-OI. This initial burst helps quickly suppress inflammation and oxidative stress around the implant, establishing an optimal biological environment for the material. Additionally, the presence of ZIF-8@BMP-9 allows for the controlled, slow release of BMP-9 through the hydrogel's degradation process, gradually delivering BMP-9 to the surrounding titanium alloy implant. This supports the integration of metal and bone interfaces, further enhancing bone repair. This sequential release mechanism effectively regulates the release of bioactive substances within the hydrogel (**Figure 1N**). At pH 7.4, the release on day 1 showed an initial burst of 41.66 ± 2.66%, likely attributed to hydrogel swelling or insufficient cross-linking in part of the network. Specifically, 4-OI released 66.15 ± 2.33% in the first 7 days, while BMP-9 released 36.92 ± 1.71% within the first 7 days, with its release rate gradually increasing to 60.74 ± 2.07% by day 28. In addition, under pH 6.5 and 8.5 conditions, with the degradation of the thermosensitive Col and molecular movement, the sustained release rate of 4-OI increased slightly, but the overall trend was consistent with that at pH 7.4 (**Figure S8A**). However, under pH 6.5, the early release rate of BMP-9 was accelerated, which may be related to the pH-responsiveness of ZIF-8 (**Figure S8B**). Changes in pH altered the degradation capacity of the thermosensitive Col, but did not change the release sequence of 4-OI and BMP-9. The early release of 4-OI plays a key role in rapidly regulating the local microenvironment around the implant, laying the foundation for subsequent bone repair. The sustained release of BMP-9 continues to promote bone integration, further enhancing the repair process. By adjusting the degradation rate and cross-linking density of the hydrogel, the sustained release of growth factors can be finely regulated, thereby extending their therapeutic effects over time.

### Biocompatibility and Hemocompatibility of Composite Scaffolds

**Figure 2:**
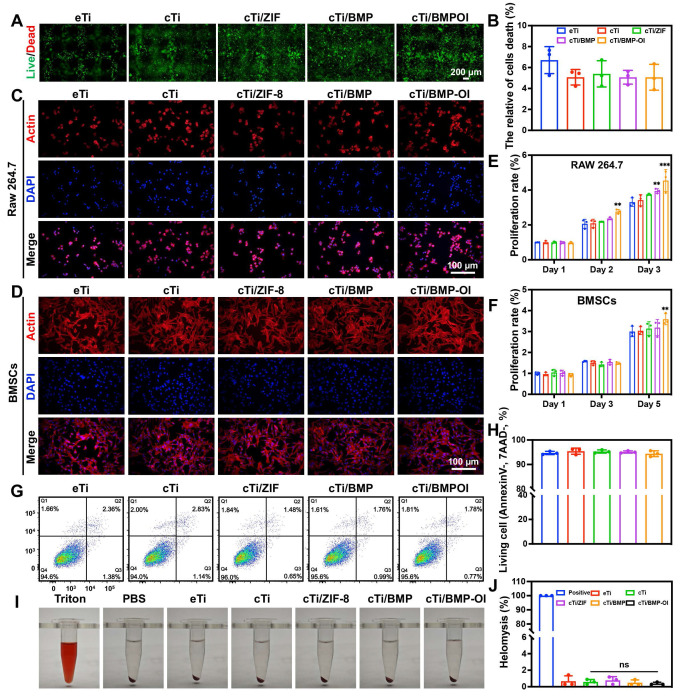
Biocompatibility of eTi, cTi, cTi/ZIF-8, cTi/BMP and cTi/BMP-OI. (A) Calcein-AM/PI staining of RAW 264.7 cells at 24 h. (B) Semi-quantitative analysis of cell viability in each group based on Calcein-AM/PI staining. (C) and (D) F-actin/DAPI staining of RAW 264.7 cells and BMSCs at 24 h. (E) and (F) Cell viability of RAW 264.7 cells and BMSCs across different groups (CCK-8 assay). (G) and (H) Percentages of living RAW 264.7 cells cultured on samples using Annexin V-PE/7AAD staining determined by flow cytometry. (I) and (J) In vitro hemolysis test of the cTi/BMP-OI composites. Images are representative of at least three independent experiments. (n = 3; data shown represent mean ± SD; *p < 0.05, **p < 0.01, ***p < 0.001, ns, no significance).

For bone tissue engineering materials, ensuring excellent biocompatibility is fundamental to achieving successful application outcomes. Therefore, this study thoroughly evaluated cell viability, proliferation, morphological changes, apoptosis, and toxicity of the composite material. Initially, RAW 264.7 cells were seeded onto scaffolds from each group and co-cultured for 24 h before performing live/dead staining. As shown in **Figure 2A**, cells successfully adhered to the scaffold surfaces. In the eTi group, which lacked a hydrogel coating, cells failed to adhere effectively to the scaffold's pores. In contrast, the other four titanium alloy scaffolds, which were coated with hydrogel, provided additional adhesion space for the cells. Staining results revealed that the number of live cells, indicated by green fluorescence, was significantly higher than the number of dead cells, marked by red fluorescence. Semi-quantitative analysis confirmed that cell viability in all groups exceeded 90% after 24 h, with no statistically significant differences between the groups (**Figure 2B**). To assess the proliferation of RAW 264.7 cells and bone marrow-derived mesenchymal stem cells (BMSCs) (**Figure S9**), Cell Counting Kit-8 (CCK-8) assays were performed (**Figure 2E-F**). The results showed a significant increase in RAW 264.7 cell numbers on days 1, 2, and 3, with a similar proliferation trend across all groups. This indicates that the materials in each group created a favorable environment for cell proliferation. In BMSC cultures, no significant variation was observed among groups during the initial two time points. At day 5, the cTi/BMP-OI group demonstrated a 1.35 ± 0.10-fold increase in proliferation relative to the eTi group, reflecting the scaffold's capacity to create a more favorable microenvironment for cellular expansion.

Phalloidin staining (FITC-Phalloidin/DAPI) was performed to further observe cell morphology. RAW 264.7 cells co-cultured with the scaffolds exhibited a relatively spherical shape, with minimal filopodia formation and no significant morphological changes after 24 h of culture (**Figure 2C**). In contrast, BMSCs co-cultured with the scaffolds showed strong cell adhesion, adopting a polygonal morphology and displaying distinct F-actin filaments. After 24 h of incubation, cell adhesion and spreading showed no marked differences between groups (**Figure 2D**). To further quantify cell viability, flow cytometry analysis using 7AAD/PE staining was performed on RAW 264.7 cells (**Figure 2G-H**). The results indicated that the average percentage of viable cells on the composite material-modified surfaces were as follows: cell viability rates for all groups were 94.6%, 94.0%, 96.0%, 95.7%, and 95.6%, respectively. Finally, a hemolysis assay was conducted (**Figure 2I**). A 1% Triton solution was used as a positive control, and physiological saline served as a negative control. After incubating red blood cells (RBCs) with the materials from each group, no significant hemolysis was observed. Furthermore, the hemolysis rate for all experimental groups was significantly below 5% (**Figure 2J**). These results confirm the excellent blood compatibility of the composite materials. Overall, the titanium alloy composite scaffold demonstrated excellent cellular biocompatibility, supporting its potential application in bone tissue engineering.

### Immunomodulatory and Antioxidant Effects in an Inflammatory Microenvironment

**Figure 3:**
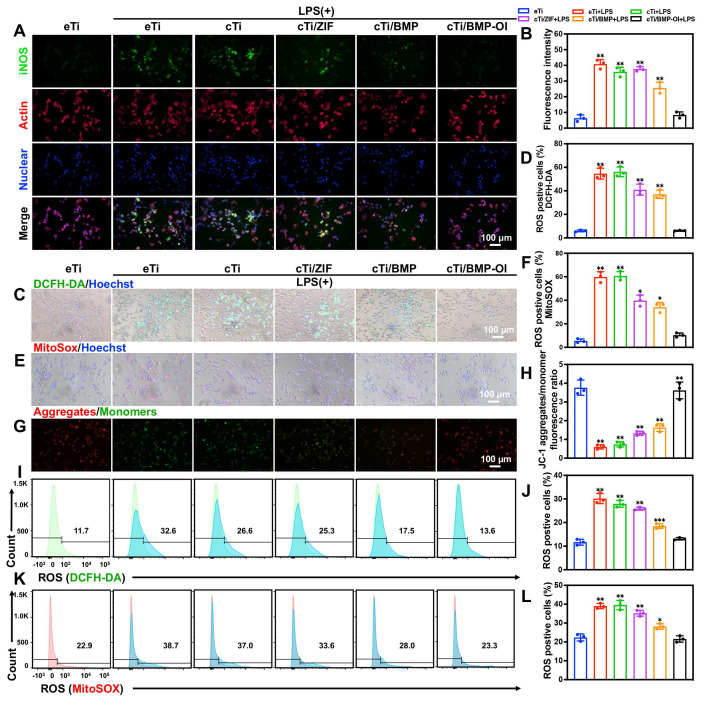
cTi/BMP-OI effectively suppresses early-stage inflammation and efficiently eliminates intracellular ROS. (A) Immunofluorescent images displaying the expression of iNOS in RAW 264.7 cells and (B) corresponding statistical analysis results for the positive. (C) and (D) Images of LPS-activated macrophages from various treatment groups stained with DCFH-DA. (E) and (F) Images of LPS-activated macrophages from various treatment groups stained with MitoSOX. (G) and (H) Representative JC-1 staining images showing mitochondrial membrane potential in LPS-stimulated macrophages under different treatments. (I) and (J) Flow cytometric analysis of DCFH-DA-positive cells. (K) and (L) Flow cytometric quantification of MitoSOX-positive cells. (n = 3; data shown represent mean ± SD; *p < 0.05, **p < 0.01, ***p < 0.001).

In osteoporotic patients, the abrupt decline in estrogen levels disrupts redox homeostasis, leading to reduced antioxidant enzyme activity and a significant increase in oxidative stress within the bone marrow microenvironment. Extensive experimental and clinical studies have demonstrated that ROS levels in the bone marrow of osteoporotic individuals are significantly higher than those in healthy controls, resulting in a high-ROS microenvironment 27, 28. Elevated ROS concentrations affect the biological behavior of bone tissue and its associated cellular components through various mechanisms, ultimately exacerbating the progression of osteoporosis. During bone regeneration, inflammation, oxidative stress, angiogenesis, and osteogenesis are closely interconnected. The initial immune response following scaffold implantation plays a pivotal role in orchestrating subsequent bone repair. Prolonged or excessive immune activation can impair bone regeneration and hinder osseointegration. One well-established immunomodulatory mechanism of biomaterials in tissue repair involves suppressing pro-inflammatory M1 macrophage polarization, thus promoting an anti-inflammatory microenvironment 29-31. Excessive accumulation of M1 macrophages contributes to increased osteoclastic activity and bone resorption, which are critical factors in implant loosening and eventual failure 32, 33. To address this, functional coatings delivering cytokines or bioactive ions have been shown to effectively suppress M1-associated inflammatory responses and improve implant-to-bone integration 34, 35. In the present study, a thermosensitive collagen-based hydrogel co-loaded with 4-OI and ZIF-8@BMP-9 was applied as a functional coating on bone implants. To explore the immunoregulatory potential of this strategy, the polarization behavior of macrophages in response to these modified surfaces was evaluated. Resting macrophages (M0) were pre-stimulated with lipopolysaccharide (LPS) to induce an inflammatory phenotype and subsequently cultured on the different material surfaces to assess their anti-inflammatory capacity. Immunofluorescence staining was utilized to visualize inducible nitric oxide synthase (iNOS), a surface marker of M1 macrophages, in RAW 264.7 cells (**Figure 3A-B**). After LPS stimulation, an increased proportion of M1 macrophages (green fluorescence) was observed on the surfaces of eTi, cTi, cTi with ZIF-8 (cTi/ZIF-8), and cTi with ZIF-8@BMP-9 (cTi/BMP) groups. In contrast, the cTi/BMP-OI group exhibited significantly lower expression of iNOS. Notably, compared to the control groups (eTi, cTi, and cTi/ZIF-8), the cTi/BMP group showed reduced iNOS expression, which can be attributed to the immunomodulatory role of BMP-9 in regulating the local bone immune microenvironment.

To assess intracellular and mitochondrial ROS levels, DCFH-DA and MitoSOX fluorescent probes were used, respectively. As shown in **Figure 3C-F**, RAW 264.7 macrophages stimulated with LPS and co-cultured with cTi/ZIF-8, cTi/BMP, or cTi/BMP-OI scaffolds exhibited a marked reduction in ROS production, with the cTi/BMP-OI group showing the most pronounced decrease—approaching the basal level observed in unstimulated control cells. Quantitative analysis was performed using flow cytometry. As shown in **Figure 3I-J**, the percentage of DCFH-DA-positive cells in the LPS-treated group was 30.17 ± 2.15%, which significantly decreased to 25.87 ± 0.60%, 18.47 ± 1.00%, and 13.10 ± 0.50% following treatment with cTi/ZIF-8, cTi/BMP, and cTi/BMP-OI scaffolds, respectively. The proportion of MitoSOX-positive cells was 39.00 ± 1.37% in the LPS group, but was reduced to 35.30 ± 1.55% in the cTi/ZIF-8 group, 28.17 ± 1.46% in the cTi/BMP group, and further to 21.57 ± 1.70% in the cTi/BMP-OI group (**Figure 3K-L**). Notably, while cTi/ZIF-8 and cTi/BMP exhibited comparable efficacy in mitigating intracellular ROS, flow cytometry revealed that cTi/BMP-OI achieved superior mitochondrial ROS clearance, likely due to a synergistic antioxidant effect between Zn^2+^ and 4-OI. Mitochondrial depolarization is associated with increased mitochondrial ROS production, which ultimately compromises mitochondrial membrane integrity 36, 37. To evaluate the ability of the cTi/BMP-OI scaffold to preserve mitochondrial membrane integrity, mitochondrial membrane potential was measured in LPS-stimulated macrophages under different treatments. JC-1, a membrane potential-sensitive fluorescent probe, was employed for this analysis. Under normal conditions, JC-1 accumulates in mitochondria, forming aggregates that emit red fluorescence. However, during membrane depolarization, JC-1 primarily exists in its monomeric form, emitting green fluorescence, a marker of increased mitochondrial permeability, apoptosis, and membrane damage. As shown in **Figure 3G-H**, LPS-stimulated macrophages exhibited a clear loss of mitochondrial membrane potential, reflected by an increased JC-1 monomer (green) to aggregate (red) fluorescence ratio. In contrast, macrophages treated with the cTi/BMP-OI scaffold displayed a significant shift toward red fluorescence, indicating a greater proportion of JC-1 aggregates and a restoration of mitochondrial membrane potential. These results suggest that the cTi/BMP-OI scaffold effectively preserves mitochondrial integrity under oxidative stress conditions. In summary, the results demonstrate that the cTi/BMP-OI scaffold mitigates oxidative damage, enhances antioxidant capacity, and alleviates ROS-induced mitochondrial dysfunction.

### Suppression of Osteoclastogenesis and Promotion of Osteogenesis

**Figure 4:**
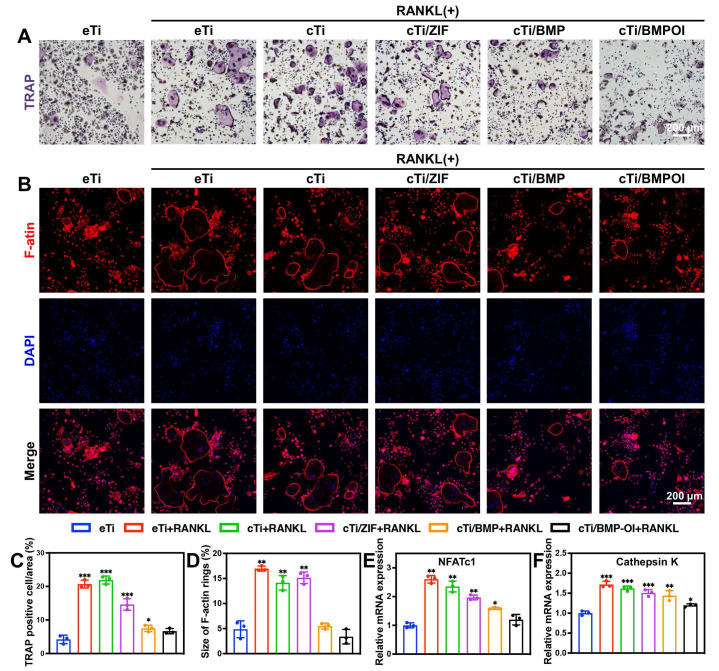
In vitro regulation of osteoblast-osteoclast coupling. (A) TRAP-stained osteoclast images with (C) corresponding quantitative analysis. (B) F-actin ring staining of osteoclasts with (D) quantitative assessment. RT-qPCR analysis of the expression levels of osteoclast-related genes (E) *NFATc1* and (F) *Cathepsin K*. (n = 3; data shown represent mean ± SD; *p < 0.05, **p < 0.01, ***p < 0.001).

BMDM cells, which serve as precursor cells to osteoclasts, were isolated from rats and co-cultured with different scaffolds to assess their influence on osteoclastogenesis. Following RANKL induction, BMDMs differentiated efficiently into osteoclasts. TRAP staining was performed to identify TRAP-positive osteoclasts in each group. As shown in **Figure 4A**, in the eTi + RANKL group, the cytoplasm of the cells exhibited a reddish or purple color, with increased cell volume, cytoplasmic extension, vacuolization, and multinucleation, which are hallmark features of mature osteoclasts. The vacuolar structure of osteoclasts is closely related to their bone resorption function. In both the eTi and cTi groups, numerous TRAP-positive osteoclasts were observed. In the cTi/ZIF-8 and cTi/BMP groups, the number of TRAP-positive osteoclasts decreased, likely due to the release of Zn^2+^ from ZIF-8, which reduced osteoclast size. In the cTi/BMP-OI group, the number of osteoclasts was significantly reduced, and some cells appeared shrunken, with reduced volume and absence of vacuolization. Random analysis of TRAP-positive cells in three different fields revealed a significantly lower number of osteoclasts in the cTi/BMP-OI group compared to the other groups (**Figure 4C**). Additionally, Clostridium perfringens toxin staining enabled the visualization of vacuolar structures in osteoclasts by marking the β-actin ring, which exhibited a significant reduction in the cTi/BMP-OI group compared to the control groups (**Figure 4B, 4D**). *Cathepsin K* is a potent osteoclastogenic factor and a critical marker of activated osteoclasts 38. *NFATc1*, a central transcription factor for osteoclast formation and differentiation, is activated by RANKL via TRAF6-NF-κB and c-Fos pathways 39. *NFATc1* activates various downstream target genes essential for osteoclast function, including those involved in cell adhesion, migration, acidification, and the degradation of inorganic and organic bone matrices 40. Additionally, *NFATc1* modulates the expression levels of *Cathepsin K* and TRAP 41. Our experimental results demonstrate that in cells co-cultured with the cTi/BMP-OI scaffold, mRNA expression levels of *Cathepsin K* and *NFATc1* were significantly downregulated (**Figure 4E-F**), suggesting that this material has the potential to inhibit osteoclast differentiation and activation.

**Figure 5:**
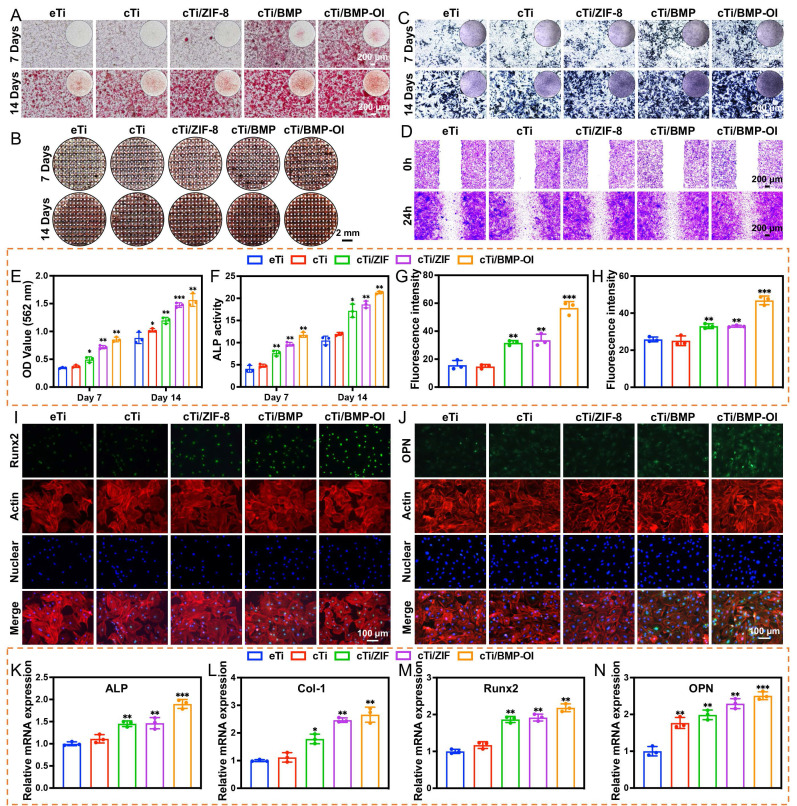
Osteogenesis induction on BMSCs. (A) and (B) Osteogenic differentiation of BMSCs assessed by ARS staining at 7 and 14 days. (C) ALP staining of BMSCs cultured with eTi, cTi, cTi/ZIF-8, cTi/BMP, and cTi/BMP-OI for 7 and 14 days. (D) Phase-contrast microscopy showing wound healing in the indicated groups. (E) Semi-quantitative evaluation of ARS staining from panel (A). (F) ALP activity assay results. (G) and (H) Quantification of fluorescence intensity for Runx2 and OPN. (I) and (J) Immunofluorescence images showing Runx2 and OPN (green), F-actin (red), and nuclei (blue). (K-N) RT-qPCR analysis of *ALP*, *Col-1*, *Runx2*, and *OPN* expression in BMSCs cultured with different scaffolds for 14 days. (n = 3; data shown represent mean ± SD; *p < 0.05, **p < 0.01, ***p < 0.001).

Osteoporosis is characterized by the destruction and thinning of trabecular bone structure, reduced bone mineral density, increased bone marrow adiposity, disrupted bone microarchitecture, and alterations in the bone matrix composition, all of which lead to a significant decline in the osteogenic potential of local bone tissue 42. The dynamic balance between osteogenesis and osteoclastogenesis is critical for effective bone tissue repair 43. To assess the impact of cTi-ZIF@BMP on the osteogenic differentiation of BMSCs, several assays were performed. Alizarin Red S (ARS) staining was used to evaluate mineralized calcium nodule formation, alkaline phosphatase (ALP) staining with quantitative analysis was applied to examine early osteogenic activity, and qPCR was conducted to determine the expression of osteogenesis-related genes, including *ALP*, collagen type I (*Col-1*), Runt-related transcription factor 2 (*Runx-2*), and osteopontin (*OPN*). Additionally, immunofluorescence staining was employed to observe the expression of Runx-2 and OPN proteins in the cells. To assess the osteogenic differentiation of BMSCs on the surface of the implants and the formation of mineralized calcium nodules in the surrounding microenvironment, ARS staining was performed on samples from all groups. On day 7 of osteogenic induction, numerous red calcified nodules were observed, particularly in the cTi/BMP and cTi/BMP-OI groups, which exhibited significantly stronger staining compared to the eTi, cTi, and cTi/ZIF-8 groups. As osteogenic induction continued, by day 14, a significant increase in calcium deposition was observed in the cTi/BMP-OI group, which showed the strongest mineralization ability (**Figure 5A**). This result is attributed to the temperature-sensitive Col in the composite scaffold, which not only provides sufficient space for cell adhesion and expansion but also facilitates the sustained release of growth factors. In contrast, the eTi scaffold, lacking the hydrogel coating, showed insufficient support for cell adhesion and differentiation due to its surface and pore structure, leading to fewer mineralized nodule formations (**Figure 5B**). To further evaluate osteogenic ability, calcium nodules located on the surface and inside the scaffold were dissolved with 10% hexadecylpyridinium solution, and the absorbance was determined by spectrophotometry. The absorbance values measured on day 7 and day 14 were significantly greater in the cTi/BMP and cTi/BMP-OI groups than in the remaining groups, indicating stronger osteogenic induction potential (**Figure 5E**). ALP is a well-established marker for early osteogenic differentiation, and its expression level serves as a reliable indicator of BMSC osteogenic differentiation status. For evaluation, samples from all groups were subjected to ALP staining following 7 days of osteogenic induction. The results indicated that the cTi/BMP and cTi/BMP-OI groups exhibited significantly stronger staining intensity compared to the eTi, cTi, and cTi/ZIF-8 groups, with a deeper reddish-purple color, indicating higher osteogenic differentiation activity. On day 14, the staining intensity in all groups increased, but the relative intensity trends remained consistent with day 7, with the cTi/BMP-OI group consistently exhibiting the strongest staining (**Figure 5C**), demonstrating its sustained osteogenic promotion effect. To confirm the findings, ALP activity was measured quantitatively with a commercial ALP assay kit. The results showed that the cTi/BMP-OI group had the highest ALP activity levels on both day 7 and day 14, significantly surpassing those of the other control groups (**Figure 5F**), further confirming the superior ability of this composite scaffold to promote early osteogenic differentiation of BMSCs. To assess the impact of the composite scaffold on the migratory ability of osteoblasts, scratch wound healing and Transwell migration assays were conducted (**Figure 5D, S10-11**). The scratch healing results showed that the cTi/BMP-OI group exhibited the highest BMSC migration ability, and the repair ability of the cTi/BMP-OI group was 2.86 ± 0.10-fold compared to the eTi group, significantly better than other experimental groups. Transwell assays indicated that BMSC migration in the cTi/BMP and cTi/ZIF-8 groups was slightly higher than in the eTi and cTi groups, but the most pronounced increase was observed in the cTi/BMP-OI group, with statistical significance. These findings demonstrate that the cTi/BMP-OI scaffold markedly promotes BMSC migration and enhances cellular activity during bone repair. Additionally, to further verify the osteogenic differentiation-promoting effect of the composite material on BMSCs, the expression levels of osteogenic-related genes, including *ALP*, *Col-1*, *Runx2*, and *OPN*, were evaluated (**Figure 5K-N**). On day 14 of osteogenic induction, gene expression in the cTi/BMP-OI group was markedly higher than in the eTi, cTi, cTi/ZIF-8, and cTi/BMP groups. Immunofluorescence staining results further supported this finding, showing a marked upregulation of Runx2 and OPN protein levels in BMSCs in the cTi/BMP-OI group (**Figure 5G-J**). In summary, the cTi/BMP-OI composite scaffold effectively promotes osteogenic differentiation of BMSCs, demonstrating significant potential for bone regeneration.

### Transcriptomic Analysis Reveals Activation of Osteogenic and Immune-Regulatory Pathways

**Figure 6:**
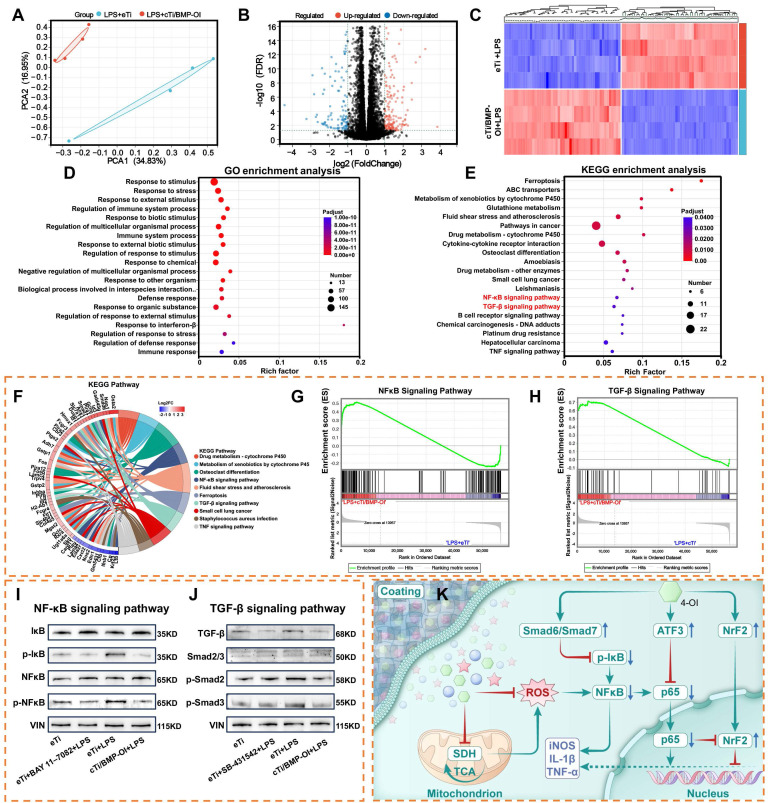
Transcriptomic analysis of the anti-inflammatory mechanism of cTi/BMP-OI. (A) PCA illustrating transcriptomic differences between the LPS+eTi and LPS+cTi/BMP-OI groups. (B) Volcano plot displaying the expression of DEGs. (C) The heat map of DEGs between LPS+eTi group and the LPS+cTi/BMP-OI group. (D) and (E) Present GO and KEGG enrichment analyses illustrating the major biological pathways and regulatory mechanisms associated with cTi/BMP-OI. (F) Top 20 KEGG enrichment chord diagram. (G) and (H) GSEA plots of NF-κB and TGF-β signaling pathways between the LPS+eTi group and the LPS+cTi/BMP-OI group. (I) Western blot bands showing expression of key NF-κB pathway regulators in different groups. (J) Western blot bands showing expression of key TGF-β signaling pathway regulators in different groups. (K) Proposed mechanism of the anti-Inflammatory effects of cTi/BMP-OI. (n = 4; data shown represent mean ± SD; *p < 0.05, **p < 0.01, ***p < 0.001).

Transcriptomic sequencing can elucidate the molecular mechanisms underlying biological pathways and disease progression, making it an invaluable tool for analyzing differential gene expression and alternative mRNA splicing. When combined with techniques such as PCR, Western blotting, and immunofluorescence, transcriptomic sequencing can effectively modulate macrophage metabolism to reduce inflammatory responses. However, the precise biological events within these metabolic processes remain incompletely understood. To further explore this, transcriptomic sequencing was performed on RAW 264.7 macrophages treated with LPS and cTi/BMP-OI, revealing distinct differences between the two groups (**Figure 6A**). Transcriptomic analysis identified 277 genes with increased expression and 167 with decreased expression in RAW 264.7 cells following treatment with cTi/BMP-OI (**Figure 6B**). The volcano plot further illustrated the distribution of inflammation-related gene markers (**Figure 6C**).

In the cTi/BMP-OI group, the expression of inflammation-associated genes including *Il12b*, *Cxcl2*, *Mmp13*, *Nos2*, and *Gsdmd* was markedly lower than in the LPS group. A gene expression heatmap visualized the patterns and trends of these differentially expressed genes. Gene Ontology (GO) enrichment analysis indicated that cTi/BMP-OI treatment significantly influenced immune system regulation, stimulus response, interspecies biological processes, response to external stimuli, and defense mechanisms (**Figure 6D**). Immune responses, cellular reactions to IFN-γ, interactions with organic substances, and responses to external stimuli were closely associated with the therapeutic effects of cTi/BMP-OI. To identify the affected signaling pathways, Kyoto Encyclopedia of Genes and Genomes (KEGG) pathway analysis was performed, revealing the top 20 enriched pathways with the lowest Q-values (**Figure 6E**). These pathways, including cytokine-cytokine receptor interactions, NF-κB signaling, TGF-β signaling, and TNF signaling, are closely related to the anti-inflammatory effects of cTi/BMP-OI. Subsequent chord diagram and Gene Set Enrichment Analysis (GSEA) confirmed the concurrent modulation of both NF-κB and TGF-β signaling pathways by cTi/BMP-OI (**Figure 6F-H**). To assess the inhibitory effects of cTi/BMP-OI on these pathways in macrophages under inflammatory conditions, Western blotting was used to evaluate the expression of key proteins. The results showed that the phosphorylation levels of NF-κB (p-NF-κB) and IκBα were lower in the presence of cTi/BMP-OI than those with LPS. Moreover, the expression levels of BAY 11-7082, an NF-κB inhibitor, were comparable to those of cTi/BMP-OI (**Figure 6I**). This suggests that cTi/BMP-OI effectively inhibits the phosphorylation activity of key proteins in the NF-κB signaling pathway, thus inhibiting the inflammatory effects of macrophages. Further analysis of the TGF-β signaling pathway revealed that LPS markedly upregulated the expression of Smad6 and Smad7 in macrophages. However, cTi/BMP-OI treatment significantly downregulated the expression of TGF-β, p-Smad2, p-Smad3, and Smad2/3. Moreover, the expression levels of SB-431542, a TGF-β inhibitor, were comparable to those of eTi and cTi/BMP-OI (**Figure 6J**). Collectively, these results suggest that cTi/BMP-OI protects LPS-stimulated macrophages from further damage by inhibiting the NF-κB signaling pathway and modulating the TGF-β signaling pathway through upregulation of Smad6 and Smad7 expression (**Figure 6K**).

The inhibitory Smad protein Smad6 plays a pivotal regulatory role at the interface between the TGF-β/BMP signaling pathway and NF-κB-mediated inflammatory responses 44. Expression of Smad6 is induced by TGF-β1 and BMP-4, and Smad6 exerts its anti-inflammatory function by directly interacting with the adaptor protein Pellino-1 within the interleukin-1 receptor/Toll-like receptor (IL-1R/TLR) signaling cascade. This interaction disrupts the formation of the interleukin-1 receptor-associated kinase 1-Pellino-1-tumor necrosis factor receptor-associated factor 6 (IRAK1-Pellino-1-TRAF6) complex, thereby preventing degradation of IκBα and subsequent nuclear translocation of NF-κB, ultimately leading to reduced expression of proinflammatory genes. Notably, knockdown of Smad6 expression abrogates the anti-inflammatory effects of TGF-β and BMP, highlighting the essential role of Smad6 in mediating these pathways 45.

Similarly, Smad7, another I-Smad, serves as a key negative regulator of the TGF-β signaling pathway and modulates its interplay with the NF-κB signaling cascade 46. Smad7 expression is transcriptionally induced by TGF-β and BMPs, constituting a classic negative feedback loop within the TGF-β signaling network 47. In addition to blocking the phosphorylation of receptor-regulated Smads (R-Smads), Smad7 inhibits NF-κB activation by promoting the transcription of IκB and interfering with the assembly of the TRAF6-TAK1-TAB2/3 complex, which is essential for NF-κB signaling. Under inflammatory conditions further induce Smad7 expression, thereby amplifying its regulatory impact on NF-κB activity. These findings underscore the dual role of Smad7 in maintaining cellular homeostasis by simultaneously restraining TGF-β and proinflammatory signaling 48.

These results suggest that cTi/BMP-OI exerts anti-inflammatory effects by suppressing pro-inflammatory pathways such as NF-κB and TGF-β, while also activating Nrf2 to enhance endogenous antioxidant production. Furthermore, cTi/BMP-OI may enhance its anti-inflammatory effect by eliminating excess ROS in inflammatory macrophages, regulating downstream pathways, and reducing pro-inflammatory molecule release.

### In Vivo Evaluation of Bone Regeneration and Osseointegration in Osteoporotic Model

**Figure 7:**
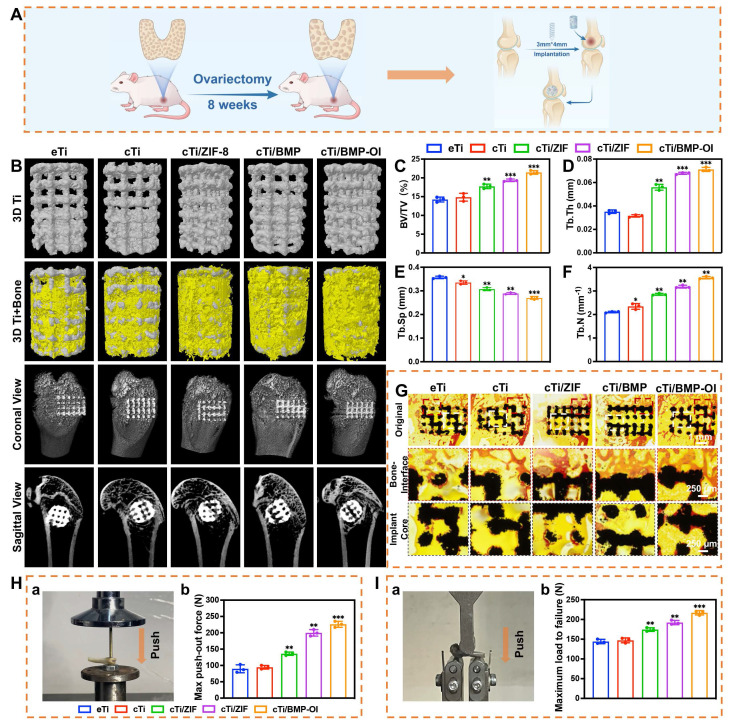
In vivo evaluation of cTi/BMP-OI scaffolds for promoting osteoporotic bone defect repair in postmenopausal rats. (A) Schematic illustrating the animal experimental protocol. (B) Representative 3D micro-CT reconstructions of scaffolds (eTi, cTi, cTi/ZIF-8, cTi/BMP, and cTi/BMP-OI) where bone tissue is shown in yellow and scaffolds in white. (C-F) Micro-CT-based quantitative indices: (C) BV/TV, (D) Tb.Th, (E) Tb.Sp, and (F) Tb.N. (G) VG staining of distal femoral defects at 8 weeks post-surgery (black: Ti6Al4V scaffold; red: new bone). (H-a) Push-out testing and (H-b) recorded peak push-out load. (I-a) Three-point bending assay and (I-b) corresponding peak load. (n = 3; data shown represent mean ± SD; *p < 0.05, **p < 0.01, ***p < 0.001).

An osteoporosis rat model was first established through ovariectomy (OVX). As shown in **Figure S12A**, bilateral OVX was performed, and two months later, the femoral distal bone of Sprague-Dawley (SD) rats was scanned using Micro-CT for three-dimensional reconstruction. The OVX group showed reduced bone mass and disrupted microarchitecture, with cortical thinning and fewer trabeculae, features typical of osteoporosis (**Figure S12B-F**). After the model was established, eTi, cTi, cTi/ZIF-8, cTi/BMP, and cTi/BMP-OI scaffolds were implanted into bone defects at the lateral femoral condyle of rats (**Figure S13A, 7A**). The rats were sacrificed at 1 and 8 weeks post-surgery, and specimens were collected for subsequent analysis (**Figure S13B-C**). Micro-CT-based three-dimensional reconstruction was employed to assess the newly formed bone around the titanium alloy scaffolds in each group (**Figure 7B**). Both coronal and sagittal views confirmed that the 3D-printed porous titanium implants were securely fixed, with no signs of loosening, displacement, or adverse bone resorption. Newly formed trabecular bone (depicted in yellow) was observed surrounding all implants, with the titanium scaffold shown in gray. Notably, the cTi/BMP-OI group exhibited significantly greater trabecular bone formation both around and within the scaffold compared to the eTi, cTi, cTi/ZIF-8, and cTi/BMP groups. Quantitative analysis of the Micro-CT data (**Figure 7C-F**) further corroborated these findings. The bone volume fraction (BV/TV) in the cTi/BMP-OI group reached 21.45 ± 0.49%, significantly higher than in the other groups. Additionally, trabecular bone metrics showed increased trabecular number (Tb.N) and decreased trabecular separation (Tb.Sp) in the cTi/BMP-OI group, consistent with enhanced bone regeneration. These findings indicate that the cTi/BMP-OI scaffold, through sequential release of bioactive agents and growth factors, enhances early osteogenic differentiation of MSCs and speeds up new bone formation. This strategy significantly enhances bone ingrowth and osseointegration of porous implants under osteoporotic conditions. To assess bone formation and osseointegration at the implant-bone interface, undecalcified hard tissue sections were prepared from samples in the eTi, cTi, cTi/ZIF-8, cTi/BMP, and cTi/BMP-OI groups. Methylene blue-acid fuchsin staining was performed to visualize bone integration. As shown in **Figure 7G**, all groups exhibited varying degrees of osseointegration between the implants and surrounding bone tissue. In the eTi, cTi, and cTi/ZIF-8 groups, sparse red-stained bone tissue was observed on the scaffold surface, with limited new bone ingrowth at the periphery and minimal osteogenesis in the central regions. In contrast, the cTi/BMP and especially the cTi/BMP-OI groups showed pronounced new bone formation, evident both at the periphery and within the central regions of the implants. These findings suggest that the cTi/BMP-OI scaffold markedly promotes bone regeneration and osseointegration of 3D-printed porous titanium implants under osteoporotic conditions, which may lower the risk of complications like implant loosening and displacement. To further evaluate the strength of osseointegration between the implant and host bone, biomechanical testing was performed by measuring the peak force required to displace the implants (**Figure 7H**). The recorded peak push-out forces for the eTi, cTi, cTi/ZIF-8, cTi/BMP, and cTi/BMP-OI groups were 89.75 ± 12.20 N, 94.47 ± 5.50 N, 135.97 ± 5.04 N, 199.85 ± 9.86 N, and 226.02 ± 9.25 N, respectively. These biomechanical results aligned with the imaging and histological assessments, confirming that the extent of new bone formation and trabecular ingrowth correlates positively with the implant's interfacial bonding strength. Furthermore, the mechanical properties of the bone surrounding the implant were assessed through a three-point bending test (**Figure 7I**). The distal femurs in the cTi/BMP-OI group exhibited a significantly higher maximum failure load—approximately 1.51 times and 1.47 times greater than those in the eTi and cTi groups, respectively. These findings provide compelling evidence that the sequential delivery of bioactive molecules and growth factors *via* the cTi/BMP-OI scaffold significantly enhances peri-implant bone mass and quality, thereby reducing the risk of implant-related fractures in osteoporotic conditions.

**Figure 8:**
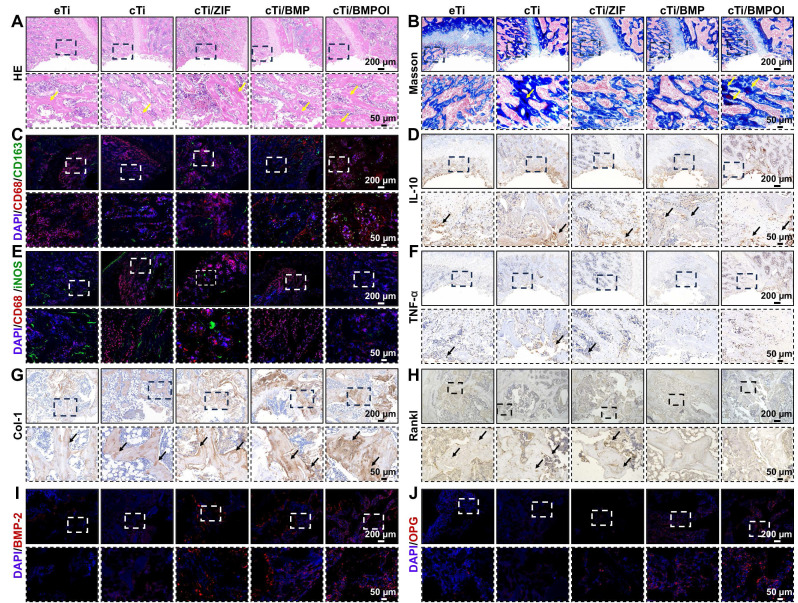
In vivo assessment of bone staining and macrophage metabolic regulation by cTi/BMP-OI. (A) HE and (B) Masson staining of bone defects at 1 week post-implantation. (C, E) Co-immunostaining of peri-implant tissue: green (M2 marker CD163; M1 marker iNOS), red (CD68, macrophage marker), and blue (nuclei). (D) and (F) Images of immunohistochemical staining of IL-10 and TNF-α in the peri-implant tissue (1 week). (G) and (H) Images of immunohistochemical staining of Col-1 and RANKL in the peri-implant tissue (8 weeks). (I) and (J) Immunofluorescence staining images of BMP-2 and OPG in peri-implant tissues (8 weeks): red (BMP-2 and OPG, osteogenic and osteoblastic marker), and blue (nuclei).

Macrophages play a pivotal role in bone immunomodulation, particularly during the inflammatory phase of early-stage bone regeneration, where their functional plasticity directly impacts healing outcomes. Suppressing the pro-inflammatory M1 phenotype is widely recognized as a key factor in promoting bone regeneration and enhancing osseointegration around implanted biomaterials. In this study, *in vitro* experiments demonstrated that the cTi/BMP-OI-modified surface exhibits superior performance characteristics, including improved cytocompatibility to support cellular proliferation, alongside a significant reduction in inflammatory responses and oxidative stress. These favorable properties provide a strong foundation for exploring the immunomodulatory capacity and osteogenic potential of the composite scaffold *in vivo*. At one week post-implantation, HE staining and Masson's trichrome staining were used to assess local inflammatory responses and early bone formation around the implant site. As shown in **Figure 8A-B**, the cTi/BMP-OI group showed reduced inflammatory cell infiltration and enhanced new bone formation, with more advanced bone maturation than the other groups. To further investigate the anti-inflammatory effects of the implants, immunohistochemical analyses were performed at the defect sites (**Figure 8C, 8E, S14A, S14C**). The cTi/BMP-OI group exhibited a lower proportion of iNOS-positive (M1 phenotype) macrophages and a higher proportion of CD163-positive (M2 phenotype) cells, indicating a favorable shift toward an anti-inflammatory macrophage profile. Given the critical roles of TNF-α and IL-10 in regulating inflammatory responses during bone healing, their expression was assessed through immunohistochemistry. The results indicated increased TNF-α expression in the eTi and cTi groups, whereas IL-10 expression was markedly elevated in the cTi/BMP-OI group (**Figure, 8D, 8F, S14B, S14D**). Collectively, these results suggest that the sequentially releasing titanium scaffold (cTi/BMP-OI) effectively suppresses early-stage inflammation and oxidative stress, facilitating early osseointegration at the bone-implant interface under osteoporotic conditions. To evaluate the in vivo balance of osteogenesis and osteoclastogenesis, immunohistochemical staining was used to detect Col-1 and RANKL, markers of bone formation and resorption, respectively. At 8 weeks post-implantation, the eTi, cTi, and cTi/ZIF-8 groups exhibited weak Col-1 staining (**Figure 8G, S14E**). In contrast, the cTi/BMP-OI group showed extensive brown and/or reddish-brown staining, indicating enhanced expression of osteogenesis-related proteins and sustained activation of matrix deposition and new bone formation. Additionally, RANKL expression was significantly higher in the eTi, cTi, and cTi/ZIF-8 groups, whereas the cTi/BMP-OI group displayed notably reduced RANKL staining (**Figure 8H, S14F**), suggesting that the cTi/BMP-OI scaffold not only promotes osteogenesis but also effectively suppresses osteoclastic activity. To further confirm these findings, immunofluorescence staining of decalcified bone sections was performed to assess osteogenic protein expression in peri-implant bone tissue. Fluorescence quantification revealed significantly elevated BMP-2 and OPG expression in the cTi/BMP-OI group compared to the eTi, cTi, and cTi/ZIF-8 groups (**Figure 8I-J, S14E-F**), consistent with the immunohistochemical results. These data collectively demonstrate that the sequential release of bioactive agents from the cTi/BMP-OI scaffold enhances osteogenic signaling while inhibiting osteoclastogenesis at the implant-bone interface under osteoporotic conditions.

Eight weeks after material implantation into the distal lateral condyle of the rat femur, histological evaluations were conducted on five major organs (heart, liver, spleen, lungs, and kidneys). HE staining (**Figure S15**) showed no obvious organ toxicity or pathological changes in the experimental groups, and all organs exhibited normal morphology comparable to the control group. In addition, HE staining and Masson staining were performed on bone tissue specimens after 8 weeks of implantation to observe potential chronic inflammatory responses around the implants. As shown in the figure (**Figure S16A-B**), the cTi/BMP-OI group showed no evident infiltration of inflammatory cells, and the inflammatory reactions in the remaining groups were also alleviated compared with those at week 1. Furthermore, immunohistochemical staining and immunofluorescence staining were used to detect inflammation-related protein (IL-1), and no significant expression of inflammatory proteins was observed around the microporous titanium alloy scaffolds (**Figure S16C**). These *in vivo* results further validate the excellent biocompatibility of the sequentially released 3D-printed titanium implants, showing no evident toxic side effects.

Osteoporosis is a systemic skeletal disease marked by decreased bone density and mass, destruction of the micro-nano architecture, and increased fragility, thereby elevating fracture risk. At the cellular level, osteoporosis primarily arises from an imbalance between osteoblast and osteoclast activity in bone tissue 49, 50. Both osteoblast and osteoclast functions are tightly regulated by the bone marrow microenvironment. ROS, including O_2_^-^, OH^-^, and H_2_O_2_, are significantly elevated at sites of osteoporotic fractures compared to normal bone tissue 51, 52. These elevated ROS levels act as negative regulators of new bone formation. Clinical studies report that H_2_O_2_ concentration at osteoporotic fracture sites is 0.5 mM higher than in healthy bone tissue 53. This may result from the substantial decline in estrogen levels in osteoporotic patients, particularly postmenopausal women, which diminishes the antioxidant capacity of bone-related cells. Consequently, ROS produced during normal physiological metabolism accumulate due to insufficient neutralization 54. Elevated ROS levels not only increase osteoclast numbers and stimulate their maturation but also inhibit the osteogenic potential of osteoblasts and MSCs, exacerbating osteoporosis 55. Additionally, the inflammatory and immune responses triggered by exogenous implants in the early stages are inevitable.

This study addresses the challenge of bone integration in osteoporotic patients by designing a titanium alloy microporous scaffold that combines immune modulation with sequential growth factor release. The goal is to modulate the immune microenvironment and enhance the integration between metal implants and bone through the use of immunosuppressants and growth factors. The proposed strategy, involving the sequential release of immune modulators and osteogenic growth factors after titanium alloy scaffold implantation, holds promise for achieving high-quality bone integration.

Compared with conventional drug-delivery strategies commonly used in bone scaffolds, our approach offers distinct conceptual and functional advantages. Traditional techniques such as surface adsorption or polymer embedding typically result in an initial burst release of bioactive agents, followed by a rapid decline in therapeutic concentration, failing to address the different physiological requirements during the sequential stages of bone healing. Moreover, these methods generally lack environmental responsiveness and do not provide coordinated control over immune modulation and osteoinduction. In this study, we designed a dual-phase sequential release system that delivers stage-specific therapeutic effects consistent with the time course of bone repair. By integrating this release profile with the structural precision of 3D-printed titanium scaffolds, the system achieves both mechanical integrity and biological functionality, offering a promising solution that surpasses the limitations of previous scaffold designs.

This study highlights the critical role of the immune microenvironment in bone repair, particularly in osteoporotic patients, where local immune responses and oxidative stress significantly influence bone healing. By releasing 4-OI, the study effectively suppressed local immune responses, notably reducing the pro-inflammatory effects of M1 macrophages, thus creating a favorable immune environment for bone repair. Additionally, the antioxidant properties of 4-OI reduced local ROS levels, further alleviating the detrimental effects of inflammation. RNA sequencing analysis revealed the specific mechanism through which the sequential release system modulates inflammation. The release of 4-OI, Zn^2+^, and BMP-9 *via* the thermosensitive Col inhibited the NF-κB and TGF-β signaling pathways, suppressing macrophage-mediated inflammation. While immunomodulation plays a pivotal role in bone repair, a single immunomodulatory approach alone is insufficient to resolve bone healing challenges in osteoporotic patients. This study, therefore, integrates the concurrent release of immunomodulators and osteogenic factors, enhancing the local immune environment while also promoting bone formation and integration through sustained BMP-9 release. Moreover, the mechanical properties of the titanium alloy scaffold are essential for effective bone repair. The study utilized 3D printing technology to fabricate titanium alloy scaffolds with high porosity and optimal pore sizes, providing the necessary structural support for bone regeneration. The results show that the scaffold's porous structure and biocompatibility create an ideal platform for cell attachment and growth. The thermosensitive Col effectively encapsulates bioactive molecules, ensuring their stability and controlled release. When combined with ZIF-8, BMP-9 was effectively encapsulated, extending its release duration *in vivo* while maintaining sustained biological activity. These design and optimization strategies not only improved the scaffold's biocompatibility but also enhanced its efficacy in promoting bone repair.

Although this study represents significant progress in enhancing the biological performance of functionalized titanium alloy scaffolds, several aspects warrant further investigation. First, more precise control over the sequential release of 4-OI and BMP-9 is required. Previous research has shown that the drug release rate directly impacts therapeutic outcomes, and both excessively fast and slow release rates can negatively affect bone healing quality. Future efforts should focus on optimizing the degradation rate of the hydrogel and refining the release characteristics of bioactive molecules to ensure that both substances are delivered at the optimal timing and dosage during bone repair. Additionally, the specific mechanisms underlying the combined use of immunomodulators and growth factors in bone repair are not fully understood. Future work should investigate how different immunomodulators and growth factors interact, and refine release strategies tailored to distinct treatment stages. Despite these challenges, this study presents an innovative solution for treating osteoporotic bone defects. By sequentially releasing immunomodulators and growth factors, bioactive molecules can be precisely delivered at different stages of treatment, significantly improving bone repair outcomes. With ongoing advancements in 3D printing technology, future research may enable the customization of personalized implants tailored to the unique needs of patients with varying bone defects. This bone repair strategy, based on the release of immunomodulators and growth factors, holds great potential for clinical application, particularly in patients with special pathological conditions such as osteoporosis, offering more effective treatment options for these cases.

## Conclusion

In conclusion, we developed a 3D-printed titanium alloy scaffold with a sequential release system, combining temperature-sensitive Col and ZIF-8@BMP-9 composites to enable controlled delivery of bioactive substances. This composite scaffold alleviates inflammation and oxidative stress within the osteoporotic microenvironment, thereby markedly improving bone defect repair. The findings highlight that the temperature-sensitive Col serves as an ideal platform for the sustained release of immunomodulators, such as 4-OI, while ZIF-8@BMP-9 ensures the long-term, continuous release of BMP-9, further promoting bone repair. Both *in vitro* and *in vivo* experiments confirm the excellent biocompatibility and immunomodulatory properties of this composite material, demonstrating substantial benefits in promoting bone integration and improving bone-metal interface bonding. Additionally, the study emphasizes the positive impact of the cTi/BMP-OI composite on macrophage polarization and ROS clearance. These results present a promising therapeutic strategy for osteoporotic patients and offer new insights and research opportunities for future clinical applications.

## Materials and Methods

### Fabrication of 3D-Printed Titanium Alloy Scaffolds

In this study, EBM technology was employed to fabricate porous titanium alloy implants using Ti-6Al-4V alloy powder as the raw material. Two types of scaffolds were designed and prepared: a disc-shaped scaffold (diameter 10 mm, height 3 mm) for physicochemical property testing and cell experiments, and a cylindrical scaffold (diameter 3 mm, height 4 mm) for mechanical property testing and animal experiments. The detailed preparation process is as follows: First, a three-dimensional STL model of the porous scaffold with a pore size of 500 μm and porosity of 70% was constructed using CAD software, with shapes designed as a disc and a cylinder. The model was then sliced using Cura software, with a layer overlap angle of 90°, generating a G-code file, which was subsequently imported into the EBM system. During the molding process, the chamber temperature was maintained at approximately 700 °C under vacuum, and Ti-6Al-4V powder was melted layer by layer using an electron beam (with a projected diameter of approximately 100 μm), completing the scaffold formation. All porous titanium alloy scaffolds were subjected to ultrasonic cleaning in acetone, ethanol, and deionized water for 60 min, repeated three times. They were subsequently sterilized in an autoclave at 121 °C for 20 min, dried in a 60 °C oven, and finally exposed to ultraviolet irradiation. The surface microstructure of the scaffolds was observed using SEM (Zeiss Sigma 300, Germany), and the porosity and pore size were determined using image analysis methods.

### Preparation of Thermoresponsive Collagen Hydrogel

The preparation of the thermoresponsive collagen hydrogel was based on a collagen solution (final concentration of 3 mg/mL, pH 3.0) extracted from pig tendons, which was further mixed with MEM concentrated medium and a buffer solution (containing sodium hydroxide, sodium bicarbonate, and HEPES) to adjust the pH and ensure the stability of the collagen as well as its subsequent gelation properties. The volume ratio of the three components was 8:1:1. At 4 °C, the components were mixed until uniform to prepare a collagen solution, which was then incubated at 37 °C for 10 min to trigger thermoresponsive gelation, converting the sol into a gel and forming a collagen hydrogel.

### Synthesis of ZIF-8 and ZIF-8@BMP-9

ZIF-8 was synthesized using a one-pot method. First, 500 mg of 2-MeIM was dissolved in 4 mL of deionized water and mixed thoroughly under magnetic stirring at room temperature. Separately, 2.5 g of zinc nitrate hexahydrate (Zn(NO_3_)_2_·6H_2_O) was dissolved in 1 mL of deionized water, and the solution was slowly added dropwise to the 2-MeIM solution while stirring continuously. During the reaction, a milky white precipitate gradually formed, indicating the generation of ZIF-8 crystals. After 30-60 min of reaction, the product was separated by centrifugation at 13,000 rpm for 20 min. The precipitate was washed three times with deionized water and then freeze-dried to obtain ZIF-8 nanoparticles. The preparation of ZIF-8@BMP-9 was based on the above method, with BMP-9 (1 µg) added to the Zn(NO_3_)_2_·6H_2_O solution. The mixture was magnetically stirred, followed by centrifugation, and the resulting supernatant was collected for encapsulation efficiency analysis. The precipitate was washed three times with water and freeze-dried to obtain ZIF-8@BMP-9 nanoparticles.

### Fabrication of Composite Scaffold with Sequential Delivery Capability (cTi/BMP-OI)

After material synthesis, disc-shaped scaffolds were placed in 48-well plates. At 4 °C, thermoresponsive collagen hydrogel was mixed with ZIF-8@BMP-9 and 4-OI (100 µM) and applied to the scaffolds, filling their pores. The constructs were then incubated at 37 °C for 10 min to form 3D-printed titanium alloy scaffolds with sequential release of 4-OI and BMP-9.

### Physicochemical Characterization of Materials

After freeze-drying, the synthesized materials were subjected to further characterization. The morphology of the samples was observed using a 20 kV SEM and TEM (JEM-F200, Japan). Elemental distribution in ZIF-8, ZIF-8@BMP-9, eTi, cTi, and cTi/BMP-OI was analyzed using EDS (JEM-F200, Japan). XRD (Rigaku, Japan) was employed to analyze the crystallographic phases of the metal-organic framework and thermoresponsive collagen hydrogel. The scanning range was from 10° to 90° 2θ, with a scanning speed of 3°/min and a step size of 0.01°. FTIR (Nexus, USA) was used to detect functional group changes in the hydrogel and metal-organic framework. The shear viscosity and elastic modulus of the hydrogel were measured using a rheometer (Kinexus, Malvern, UK). The hydrated particle size of ZIF-8 and ZIF-8@BMP-9 was determined by DLS (Malvern Zetasizer Nano ZS, Germany).

### Assessment of Cell Proliferation by CCK-8 Assay

Cell proliferation ability was assessed using the CCK-8 assay. RAW 264.7 cells and BMSCs were seeded in 24-well plates at a density of 1 × 10⁴ cells per well. RAW 264.7 cells were evaluated on days 1, 2, and 3, while BMSCs were assessed on days 1, 3, and 5. Before testing, the medium was replaced with fresh medium containing 10% CCK-8 solution (1:10 dilution). The cells were incubated at 37 °C with 5% CO_2_ for 2 h. Subsequently, 100 μL of the supernatant from each well was transferred to a 96-well plate, and absorbance at 450 nm was measured with a microplate reader to assess cell metabolism and proliferation.

### Flow Cytometry-Based Apoptosis Analysis

Cell viability and apoptosis were evaluated by Annexin V-PE/7-AAD double staining and flow cytometry. Scaffolds from each group were placed in 6-well plates, co-cultured with RAW 264.7 cells for 24 h, and the cells were then harvested for analysis. The cells were first washed twice with pre-chilled PBS, with centrifugation at 1,800 rpm for 5 min at 4 °C each time. The supernatant was discarded. Subsequently, 100 μL of 1× Binding Buffer was added to the cell pellet, and the cells were gently resuspended to form a single-cell suspension. Next, 5 μL of Annexin V-PE and 5 μL of 7-AAD staining solution were added, mixed gently, and incubated at room temperature in the dark for 10 min. After incubation, 400 μL of 1× Binding Buffer was added to mix the cells. The stained samples were then immediately analyzed by flow cytometry. FlowJo software was used to analyze the data, distinguishing early apoptosis (Annexin V⁺/7-AAD⁻), late apoptosis (Annexin V⁺/7-AAD⁺), and necrosis (Annexin V⁻/7-AAD⁺).

### RNA Sequencing for Transcriptomic Analysis

RNA sequencing (RNA-seq) was used to examine gene expression in macrophages co-cultured with composite scaffolds under LPS stimulation. Briefly, RAW 264.7 macrophages were seeded in 6-well plates, and after cell attachment, they were treated with 100 ng/mL LPS for 8 h to induce an inflammatory phenotype. The culture medium was then replaced, and the eTi and BMP/OI composite scaffolds were placed into the wells to co-culture with the cells for 48 h. Total RNA was isolated from each cell group with TRIzol reagent and stored at -80 °C for subsequent use. RNA sequencing was conducted by the majorbio platform (https://www.majorbio.com/), and the resulting transcriptomic data were analyzed for enrichment and functional annotation using GO and KEGG pathway analyses.

### Establishment of Osteoporotic Bone Defect Model in Rats

All animal procedures followed the NIH guidelines for the care and use of laboratory animals (Publication No. 8023, revised 1978) and received approval from the Ethics Committee of Shanghai Tenth People's Hospital, Tongji University School of Medicine (Approval No. SHDSYY-2024-Y3789). Model Construction: An osteoporosis model was established in female SD rats using bilateral OVX. Forty female Sprague-Dawley rats (8 weeks old, ~200 g) were enrolled, with 36 assigned to the OVX group and 4 to the Sham group. Animals were fasted and water-deprived for 12 h prior to surgery, and anesthesia was induced by intraperitoneal injection of 3% pentobarbital sodium. After disinfecting the surgical field, a small incision was created 1-2 cm lateral to the spine beneath the final rib, enabling exposure of the ovaries. The associated vessels and pedicles were ligated, and both ovaries were excised. In the Sham group, the ovaries were only exposed without removal. Post-surgery, all animals received subcutaneous injections of penicillin (2 mg/kg) for 3 consecutive days, and the surgical sites were disinfected daily. After surgery, the rats were allowed to move freely. At the 8-week post-surgery mark, 4 rats were euthanized, and femur and other bone tissue samples were collected to evaluate the establishment of osteoporosis, including analysis of bone mineral density (BMD) and trabecular bone structure.

Rats were deprived of food and water for 12 h before being anesthetized with 3% pentobarbital sodium administered intraperitoneally. Once anesthesia was achieved, the hair around the long axis of the right femur was shaved, and the skin over the femoral condyle and knee joint was thoroughly exposed. The osteoporosis model rats were then positioned in a left lateral decubitus position on the surgical table, with the right knee joint area exposed. The area was disinfected using iodine tincture, followed by the application of a sterile drape. Subsequently, an incision was made sequentially through the skin and fascia, and blunt dissection was performed along the intermuscular planes to expose the femoral condyle. The surrounding soft tissue was carefully dissected to fully expose the lateral femoral condyle. Using a power drill, a cylindrical bone defect with a diameter of 3 mm and depth of 4 mm was prepared in the femoral lateral condyle, followed by the implantation of a scaffold implant of corresponding size. The surgical area was then closed in layers, suturing the muscle and skin. After surgery, rats received intramuscular penicillin (2 mg/kg) for 3 days, and the incision was disinfected daily with iodine tincture until complete healing. At the 1-week and 8-week postoperative time points, the animals were euthanized, and femur samples from the right implanted region were collected. Some samples were fixed in 4% paraformaldehyde, while others were stored at -80 °C for subsequent analysis.

### Micro-CT Evaluation of Osseointegration and Bone Regeneration

Micro-CT scanning (Skyscan 1275, Bruker, Belgium) was used to characterize the scaffold and adjacent bone. Reconstructed 3D images were analyzed for BMD, BV/TV, Tb.N, trabecular thickness (Tb.Th), and Tb.Sp, providing indicators of scaffold-bone integration.

### Statistical Analysis

All quantitative data are presented as mean ± standard deviation (SD). Multi-group data were analyzed by one-way ANOVA, and intergroup differences were further examined using Tukey's post hoc test. Statistical calculations were carried out in GraphPad Prism 9, with p values below 0.05 regarded as statistically significant. Statistical significance was denoted as follows: *p < 0.05, **p < 0.01, ***p < 0.001.

## Supplementary Material

Supplementary materials and methods, figures and table.

## Abbreviations

4-OI: 4-octyl itaconate
BMP-9: bone morphogenetic protein-9
BMSC: bone marrow mesenchymal stem cell
ROS: reactive oxygen species
MOFs: Metal-organic frameworks
ZIF-8: Zeolitic imidazolate framework-8
SEM: scanning electron microscopy
TEM: transmission electron microscopy
EDS: energy-dispersive X-ray spectroscopy
DLS: dynamic light scattering
ELISA: enzyme-linked immunosorbent assay
FTIR: fourier-transform infrared spectroscopy
XRD: X-ray diffraction
LPS: lipopolysaccharide
iNOS: inducible nitric oxide synthase
Col-1: collagen type I
Runx-2: runt-related transcription factor 2
OPN: osteopontin
BV/TV: bone volume fraction
Tb.N: trabecular number
Tb.Sp: trabecular separation
Tb.Th: trabecular thickness
