# Supplementary material for: Sequential release of bioactive factors from functionalized metal-organic framework hydrogel enhances interfacial osseointegration of 3D-printed titanium alloy porous scaffolds
Source: Theranostics. 2026 Jan 1;16(2):852–75. doi: 10.7150/thno.120711 (PMC12674936; doi:10.7150/thno.120711)
Supplement: Supplementary file 1 — Supplementary materials and methods, figures and table. [file thnov16p0852s1.pdf]

Sequential release of bioactive factors from functionalized metal–organic framework hydrogel enhances interfacial osseointegration of 3D-printed titanium alloy porous scaffolds

Zhenjia Che[a1], Xiao Sheng[b1], Yanglin Wu[a], Binghao Lin[a], Kaihang Song[a], Qiyun Chen[a], Aopan Chen[a], Lingxuan Deng[a], Jing Chen[a], Ming Cai[a]*

a Department of Orthopaedics, Shanghai Tenth People's Hospital, School of Medicine, Tongji University, No.301 Middle Yanchang Road, Shanghai 200072, P. R. China
b Huzhou Central Hospital, Fifth School of Clinical Medicine of Zhejiang Chinese Medical University, Wuxing, Huzhou, Zhejiang 313000, P. R. China

* Corresponding author: Ming Cai: cmdoctor@tongji.edu.cn

Zhenjia Che and Xiao Sheng contributed equally to this work.

Corresponding author: Ming Cai (cmdoctor@tongji.edu.cn) will handle correspondence at all stages of refereeing and publication, also post-publication.

Materials and methods

Materials

All chemicals were sourced from commercial suppliers and used without further purification. RAW 264.7 cells were obtained from Biotime Biotechnology (Shanghai, China). DMEM, F-12 medium, fetal bovine serum (FBS), trypsin, and penicillin–streptomycin were purchased from Gibco (USA). $Zn(NO_3)_2 \cdot 6H_2O$ and 2-methylimidazole were sourced from Macklin Biochemical Technology (Shanghai, China), while type I collagen (Col-1) and 4-OI were obtained from Corning (USA) and MedChemExpress (USA), respectively. Recombinant BMP-9 was provided by PeproTech (USA), and BMP-9 ELISA kits were supplied by Biodesign International (China). Functional assay kits, including Alizarin Red S, CCK-8, live/dead staining, ALP, and crystal violet, were purchased from Biotime Biotechnology (Shanghai, China). Annexin V-PE/7-AAD apoptosis detection kits were acquired from Vazyme Biotech (China), whereas RANKL, M-CSF, and TRAP staining kits were purchased from Mingyang Biotech (China). Yeasen Biotech (Shanghai, China) provided the QuantiNova SYBR PCR Mix and RNA extraction kits. Antibodies against Runx2, OPN, and iNOS, together with secondary antibodies, were obtained from Abcam (UK). FITC-CD206 and APC-CD86 were sourced from BioLegend (USA). Experimental Sprague–Dawley rats were obtained from Slake Biotech (China).

Encapsulation Efficiency

To determine the encapsulation efficiency of BMP-9 in ZIF@BMP-9, the supernatant collected after centrifugation was used as the mother liquor. The encapsulation efficiency (EE) was calculated using an enzyme-linked immunosorbent assay (ELISA). The formula for calculating the BMP-9 encapsulation efficiency (EE) is as follows:

$$\mathrm{EE}(\%) = [\ (\text{total BMP-9}) - (\text{BMP-9 in mother liquor}) / \text{total BMP-9}\ ] \times 100$$

Swelling Ratio and Degradation Behavior of Thermoresponsive Collagen Hydrogel

Dry hydrogel weight (W0) was recorded, followed by immersion in PBS at 37 °C with hourly buffer replacement. At each interval, samples were removed, blotted, and weighed (Wt) until equilibrium was established. ESR was calculated using the equation:

$$\mathrm{ESR} = (\mathrm{Wt} - \mathrm{W0}) / \mathrm{W0} \times 100\%$$

Here, Wt and W0 correspond to the swollen and dry weights of the hydrogel,

respectively, and all measurements were performed in triplicate.

The degradation profiles of the thermoresponsive collagen hydrogel and its functionalized counterpart were examined under pH 6.6, 7.4, and 8.5. Disc-shaped samples (10 mm in diameter, 2 mm thick) were first weighed (W0) and then immersed in the respective solutions. At predetermined intervals, samples were taken out, blotted dry, weighed (Wd), and recorded. The degradation rate (DS) was then determined using the following formula:

$$DS = (W0 - Wd) / W0 \times 100\%$$

The release of 4-OI and BMP-9 from the thermoresponsive collagen hydrogel was quantified using UV spectrophotometry and ELISA, respectively. Composite hydrogels were placed in 12-well plates and incubated at 37 °C with 2 mL of solutions under different pH conditions. Medium sampling was performed at scheduled intervals (1 h, 3 h, 8 h, 1, 3, 7, 10, 15, 21, 28 days), after which the same volume of fresh solution was introduced. The concentrations of 4-OI and BMP-9 were determined, with 4-OI measured at its maximum absorption wavelength of 199 nm. Standard curves were used to calculate cumulative release percentages.

Rheological Testing

The rheological behavior of the hydrogel, including elastic modulus and viscosity, was characterized using a rheometer. Samples were placed on a parallel plate (25 mm diameter) with a 0.4 mm gap. In stress mode, tests were carried out at 25 °C under an angular velocity of 6.28 rad/s, with a frequency of 1 Hz and strain ranging from 0.1% to 1000%. Storage modulus (G′) and loss modulus (G″) were recorded. In shear rate mode, measurements were conducted at 25 °C to determine shear viscosity and shear stress across a shear rate range of 0.1–1000 s^{-1}.

Cell Culture

Primary BMSCs were isolated from SD rats aged 6–8 weeks. Following euthanasia, tibiae and femora were excised, disinfected in 75% ethanol for 15 min, and stripped of residual tissue. Bone marrow was extracted with a 1 mL syringe, centrifuged, and treated with red blood cell lysis buffer on ice. The cell pellet was resuspended in low-glucose DMEM containing 10% FBS and 1% penicillin–streptomycin, and maintained at 37 °C in 5% CO_2. When cell confluence reached ~80%, digestion with 0.25% trypsin was performed, followed by subculture, with third-generation cells used for experiments.

Primary BMDMs were isolated following a procedure analogous to that of BMSCs. Bone marrow from the hind limbs of 6–8-week-old mice was harvested, centrifuged, and subjected to red blood cell lysis, then centrifuged again. The pellet was resuspended in DMEM supplemented with 50 ng/mL M-CSF and cultured for 24 h before being transferred to flasks for an additional 48 h. Cells that adhered were designated as BMDMs. These were seeded into 24-well plates and maintained in DMEM containing 50 ng/mL M-CSF and 50 ng/mL RANKL. TRAP staining performed on day 4 verified the presence of multinucleated osteoclasts.

RAW264.7 cells were maintained in high-glucose DMEM and subsequently passaged for later experiments.

Cell phenotype identification by flow cytometry

Flow cytometry was used to identify BMSC surface markers. Cells were fixed in 50% ethanol for 30 min, centrifuged, and resuspended to form a 100 μl single-cell suspension. Cells were first incubated with primary antibodies to CD31, CD44, CD45, and CD90 for 30 min, then exposed to FITC-labeled secondary antibodies for another 30 min at 4 ℃ in darkness. After washing with PBS, the cells were resuspended and subjected to flow cytometric analysis.

Cell Viability Assessment

Cell viability of RAW 264.7 cells was evaluated using a Live/Dead Cell Staining Kit. Scaffolds from each group were placed in 48-well plates, seeded with RAW 264.7 cells, and co-cultured for 24 h. After incubation, the medium was removed, and wells were washed three times with PBS to eliminate residual medium and non-adherent cells. The working solutions for live and dead cell staining were prepared according to the kit's instructions. Initially, an adequate volume of live-cell dye was applied to fully cover the cells and incubated at 37 °C for 30 min under dark conditions. After incubation, wells were rinsed with PBS to eliminate non-specific staining and reduce background signals. Subsequently, an equal volume of the dead cell dye was added, and incubation was continued under the same conditions for 20 min, followed by another PBS wash. After staining, cell viability was observed and imaged using a fluorescence microscope. The maximal emission wavelength for live cells was 515 nm (green), while the maximal emission wavelength for dead cells was 617 nm (red).

Effect of Different Coating Strategies on BMSC Adhesion Morphology

To observe the adhesion morphology and cytoskeletal structure of RAW 264.7 cells and BMSCs on different scaffold surfaces, scaffolds from each group were placed in 24-well plates. A total of 2×10^4 RAW 264.7 cells and BMSCs were seeded in each well and incubated at 37°C with 5% CO_2 for 24 h, followed by cytoskeletal and nuclear staining. After discarding the medium, cells were rinsed with PBS, fixed in 4% paraformaldehyde for 10 min, and then permeabilized using 0.5% Triton X-100 for 10 min. After rinsing, cytoskeletal F-actin was visualized using 100 nM Phalloidin–Alexa Fluor staining for 30 min in darkness, and nuclei were counterstained with 100 nM DAPI for 30 s. The specimens were then PBS washed and imaged with a fluorescence microscope.

Hemolysis Test

Tail vein blood (1 mL) was collected from rats, centrifuged at 1000 rpm to discard plasma, and the erythrocytes were washed three times with saline. A 2% suspension was prepared by resuspending the pellet in saline. This suspension (500 μL) was mixed with 500 μL scaffold extract, incubated at 37 °C for 3 h, and centrifuged for 5 min at 1000 rpm. Physiological saline was used as the negative control, and 5% Triton X-100 was applied as the positive control. After incubation, 200 μL of the supernatant was pipetted into a 96-well plate, and absorbance was measured at 547 nm using a microplate reader. Hemolysis percentage was subsequently calculated using the standard equation:

Hemolysis rate (%) = (OD of experimental group / OD of pure water group) × 100%

Macrophage Inflammation Inhibition and ROS Clearance

To assess macrophage anti-inflammatory activity, immunofluorescence staining was conducted. RAW 264.7 macrophages were cultured on cell-coated slides in 24-well plates, exposed to 100 ng/mL LPS for 8 h, and subsequently co-cultured with extracts from different material groups for 24 h. Cells were fixed and treated with 5% goat serum for blocking, then incubated overnight at 4 °C with the iNOS primary antibody. After rinsing, they were exposed to a fluorescent secondary antibody for 1 h at room temperature. Nuclear staining was carried out with DAPI, and fluorescence images were obtained using an inverted microscope.

DCFH-DA Assay

Intracellular ROS in RAW 264.7 macrophages was assessed using the DCFH-DA probe. Cells were seeded in 24-well plates, allowed to adhere, stimulated

with 100 ng/mL LPS for 8 h, and then co-cultured with scaffold extracts for 24 h. Afterward, cells were incubated with serum-free medium containing 10 μM DCFH-DA at 37 °C for 20 min to label ROS. After incubation, nuclei were counterstained with Hoechst 33342, followed by three PBS washes. Fluorescence images were captured with an inverted microscope, and ROS-positive cells were quantified using ImageJ. ROS levels were further analyzed by flow cytometry (Ex 488 nm, Em 520 nm), and results were processed with FlowJo.

MitoSox Assay

The generation of mitochondrial ROS was evaluated using the MitoSOX Red fluorescent probe. RAW 264.7 macrophages were seeded into 24-well plates and maintained until firm attachment was established. After adhesion, RAW 264.7 cells were treated with 100 ng/mL LPS for 8 h, then co-cultured with scaffold extracts for 24 h. Cells were incubated with 5 μM MitoSOX Red in serum-free medium at 37 °C for 10 min, counterstained with Hoechst 33342, washed with PBS, and analyzed for mitochondrial ROS using fluorescence microscopy and flow cytometry.

JC-1 Assay

Mitochondrial membrane potential changes were evaluated using the JC-1 mitochondrial membrane potential assay kit. RAW 264.7 macrophages were cultured in 24-well plates until adhesion, then exposed to 100 ng/mL LPS for 8 h, followed by 24 h co-incubation with scaffold extract solutions from each group. Subsequently, cells were incubated with JC-1 working solution at 37 °C for 20 min, washed twice with staining buffer, and observed under an inverted fluorescence microscope. The red/green fluorescence ratio was semi-quantified using ImageJ software.

In Vitro Inhibition of Osteoclastogenesis

BMDMs were cultured with scaffold extracts and induced to differentiate in DMEM containing 50 ng/mL M-CSF and 50 ng/mL RANKL for 4 days. Osteoclast formation was assessed by TRAP staining: after medium removal, cells were washed with PBS, fixed in pre-cooled TRAP fixative for 1 min, rinsed with distilled water, and incubated in TRAP working solution at 37 °C for 1 h. After staining, the cells were washed again with distilled water, followed by nuclear counterstaining with hematoxylin for 3 min. The cells were then rinsed with tap water for 10 min to return the blue color. The TRAP-positive multinucleated osteoclasts were observed under a microscope to assess their morphology. In this experiment, after the cells from each group were fixed, they were treated with 0.1% Triton-X permeabilization solution for

15 min and washed with PBS. Subsequently, cells were stained with FITC-conjugated phalloidin for 60 min to visualize F-actin filaments. After DAPI counterstaining, the cells were imaged under a fluorescence microscope. To further assess osteoclast differentiation at the molecular level, RT-qPCR was conducted to quantify the expression of *NFATc1* and *Cathepsin K* in osteoclasts after 4 days of co-culture. Primer sequences are provided in Table S1.

Alizarin Red S (ARS) Staining

In 24-well plates, BMSCs were cultured at a density of 1×10^4 cells per well in osteogenic medium for 7 or 14 days. After fixation in 4% paraformaldehyde and PBS washing, cells were stained with Alizarin Red to detect mineralized nodules. Scaffolds seeded with BMSCs under identical conditions in 48-well plates were processed the same way. Calcium deposits were dissolved with 10% cetylpyridinium chloride (500 μL), and absorbance at 610 nm was measured for quantification.

Alkaline Phosphatase (ALP) Staining and Activity Assay

In 24-well plates, BMSCs were grown on scaffolds to 70–80% confluence, followed by osteogenic induction for 7 or 14 days. After PBS washing and 4% paraformaldehyde fixation (30 min), osteogenic differentiation was assessed via ALP staining and activity assays. Staining was performed using a BCIP/NBT kit, where working solution was applied until color appeared, followed by termination with stop solution. For activity assays, cell lysates were prepared with IP buffer, centrifuged, and the supernatant was incubated with detection buffer at 37 °C. Absorbance at 405 nm was measured to calculate ALP activity.

Immunofluorescence and PCR

BMSCs cultured on scaffolds for 7 days were processed for immunofluorescence, including fixation, blocking with 5% goat serum, incubation with Runx2 and OPN primary antibodies at 4 °C, fluorescent secondary antibody labeling, and DAPI nuclear staining. Imaging was conducted using an inverted fluorescence microscope. Additionally, qRT-PCR was employed to assess osteogenic gene expression (*ALP*, *Runx2*, *Col-1*, and *OPN*). RNA was extracted, reverse transcribed with the HiScript III RT SuperMix kit, and amplified with primers listed in Supplementary Table S1.

Histological Evaluation (Hard Tissue Sectioning)

At 8 weeks post-surgery, samples were fixed, dehydrated through graded ethanol, cleared with xylene, embedded in paraffin, sectioned using a hard tissue microtome,

and stained with Van Gieson (VG) to evaluate bone ingrowth and scaffold–bone integration.

Push-out mechanical test

An electronic universal testing machine was used to record the maximum force needed to push the scaffold out of the distal femur, which reflected the strength of the implant–bone interface. The femoral distal medial and lateral sides were appropriately polished, and the scaffold was exposed at the medial condyle. The femoral medial condyle was fixed onto the testing platform, ensuring the implant's longitudinal axis was perpendicular to the surface of the platform. A metal plunger with a diameter of 3 mm was mounted on the testing machine, aligned vertically with the upper surface of the scaffold, and applied at a speed of 1 mm/min from the lateral condyle direction. The force applied by the plunger during the test was recorded until the implant was completely ejected, and the maximum ejection force was extracted as the mechanical parameter. Statistical analysis was then conducted on the maximum force values for each group of samples.

Three-Point Bending Test

A three-point bending test was carried out on the distal femur to assess peri-implant bone strength. Samples were positioned on a 30 mm span saddle support with the implant beneath the loading point. Using an electronic universal testing machine at 2 mm/min, vertical force was applied until fracture, and mechanical parameters were recorded.

Immunohistochemistry and Fluorescence Staining

To evaluate inflammation, osteogenesis, and macrophage polarization, femoral tissues obtained one week after implantation were fixed, decalcified, embedded, and sectioned. HE and Masson staining were carried out to examine local inflammatory infiltration and bone regeneration. For immunohistochemistry, endogenous peroxidase was inactivated with 3% hydrogen peroxide, and goat serum was applied to reduce nonspecific binding. The sections were then incubated overnight at 4°C with primary antibodies specific to macrophage polarization markers (iNOS and CD163). On the following day, corresponding secondary antibodies were applied, and the staining was completed for histological observation. Additionally, femoral samples from rats euthanized at 8 weeks post-surgery were analyzed using the same method to assess osteogenesis-related proteins (Col-1 and BMP-2) and osteoclast-related proteins (OPG and RANKL), in order to evaluate the balance between osteogenesis

and osteoclastogenesis during the bone remodeling process.

In vivo Chronic Inflammatory Response and Biocompatibility Assessment

At 8 weeks post-implantation, rats were sacrificed under anesthesia, and the heart, liver, spleen, lungs, and kidneys were harvested. These organs were fixed, embedded, sectioned, and subjected to HE staining for histological examination of potential scaffold-related toxicity. Tissue morphology was observed microscopically to evaluate systemic safety. Concurrently, femoral specimens collected at 8 weeks were processed for HE, Masson, and immunohistochemical staining to investigate inflammatory responses around the titanium alloy scaffold. Collectively, these assays demonstrated favorable biocompatibility and in vivo safety of the 3D-printed scaffold with sequential release properties.

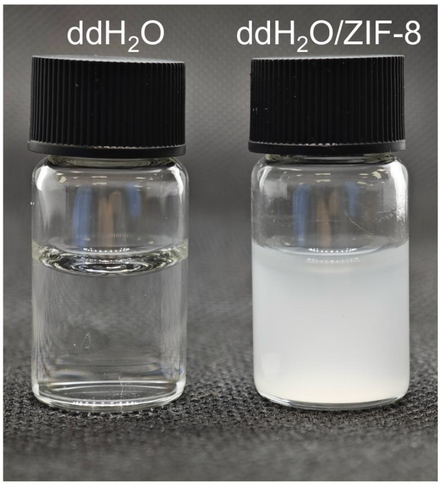

Figure S1. ZIF-8 suspended in ddH_2O.

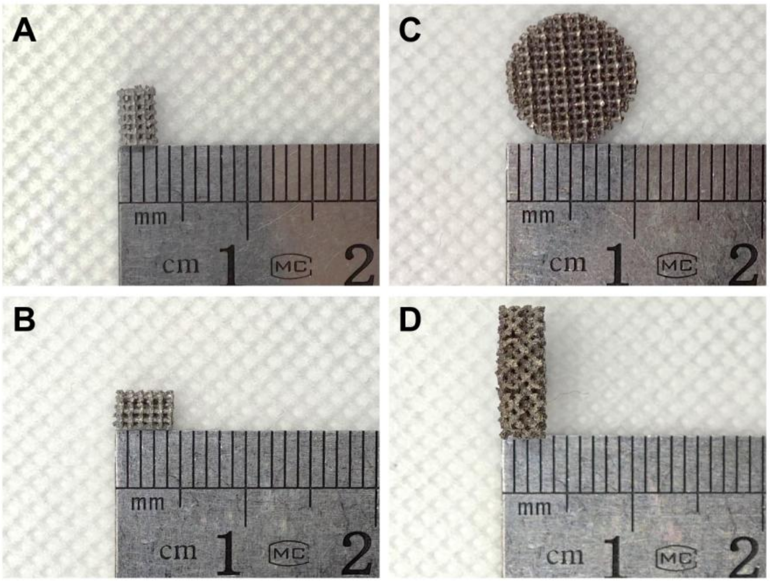

Figure S2. Characterization of disc-shaped and cylindrical eTi.

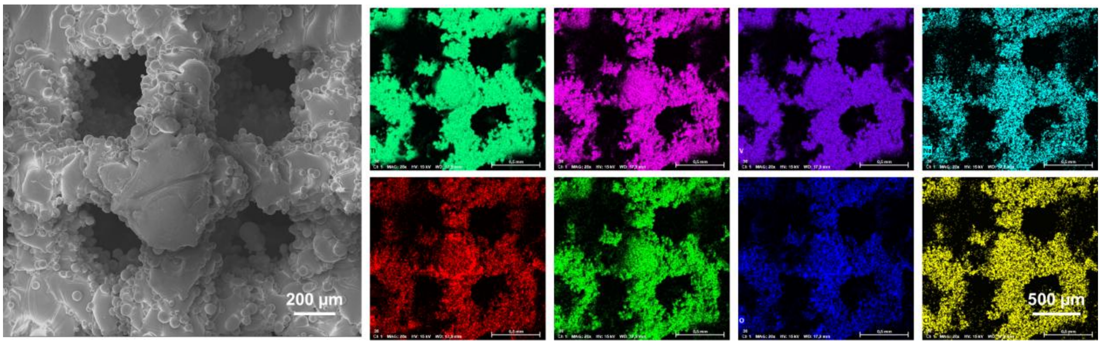

Figure S3. SEM and EDS of eTi.

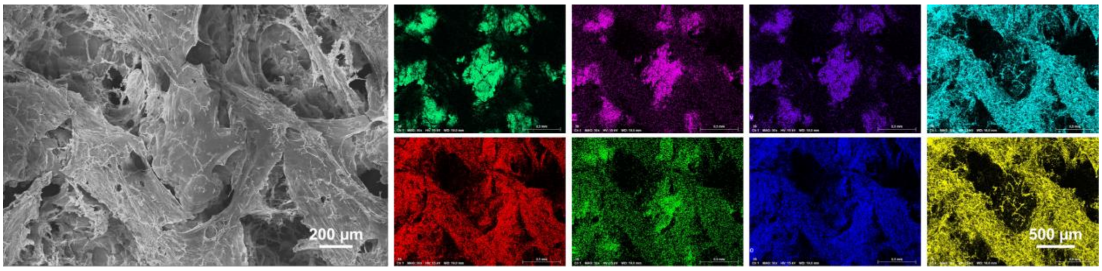

Figure S4. SEM and EDS of cTi.

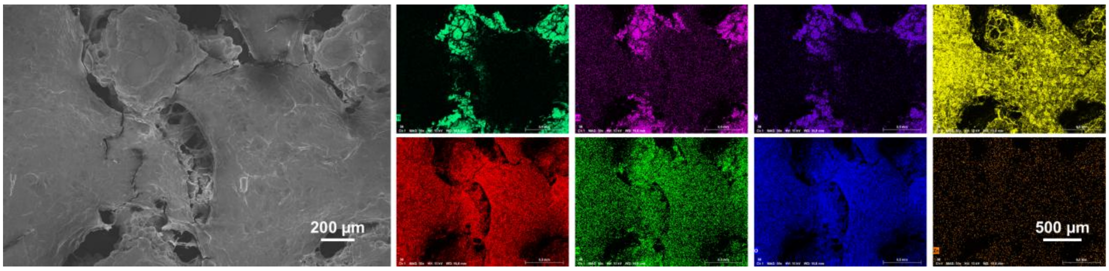

Figure S5. SEM and EDS of cTi/BMP-OI.

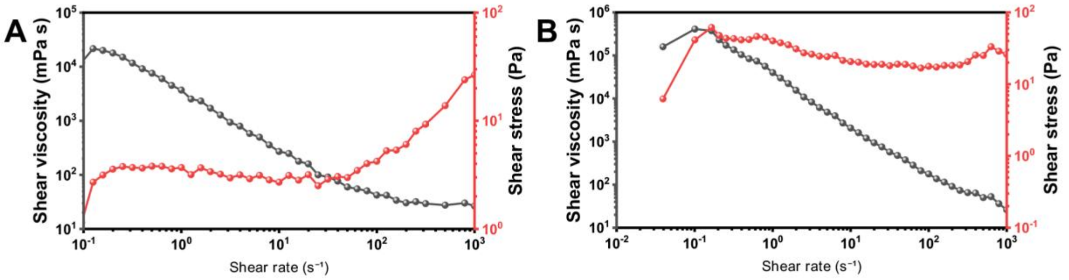

Figure S6. (A) and (B) The plot of shear stress and viscosity against shear rate for Col and Col/BMP-OI.

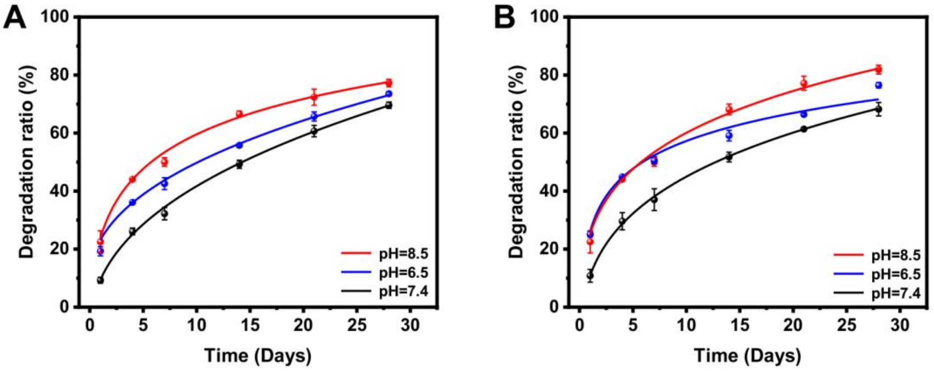

Figure S7. (A) and (B) Degradation curves of Col and Col/BMP-OI at different pH values.

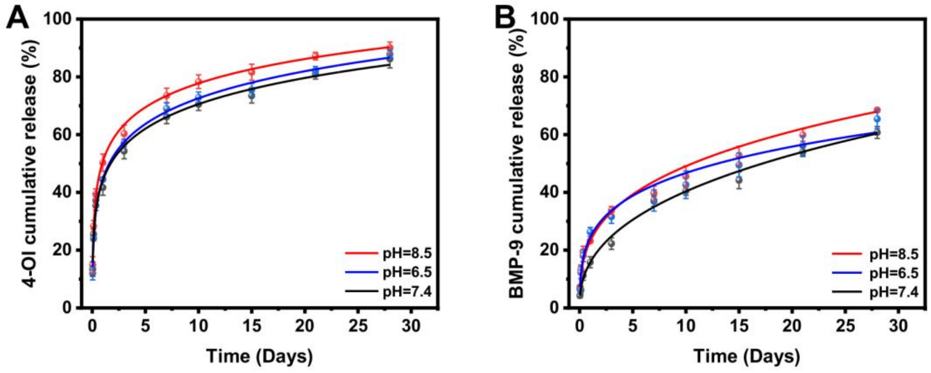

Figure S8. (A) and (B) 4-OI and BMP-9 release from cTi/BMP-OI over 28 days at different pH values.

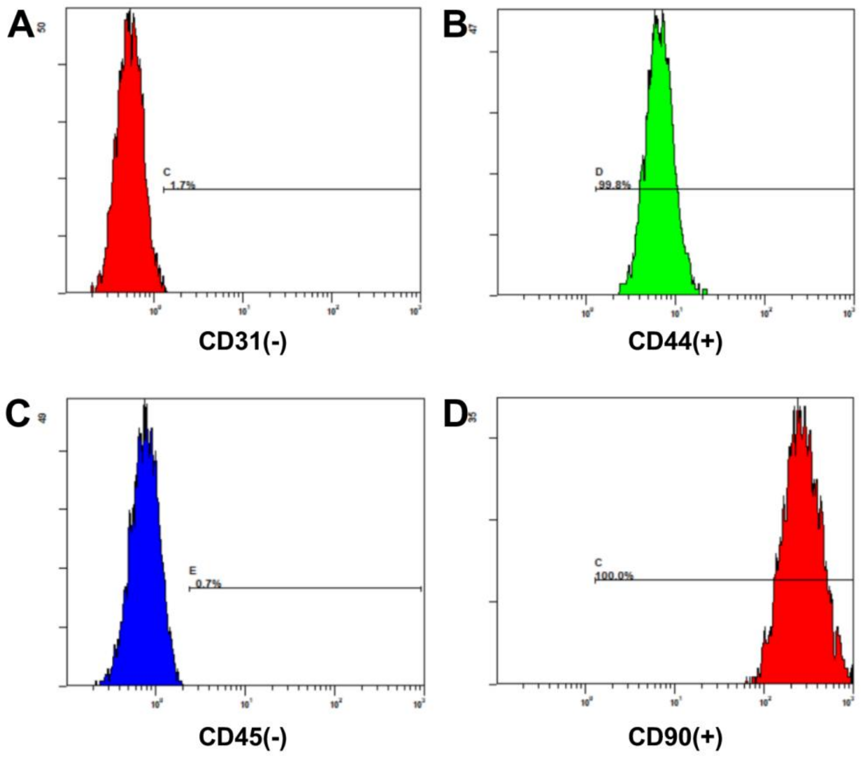

Figure S9. Flow cytometric aAnalysis of BMSC surface markers (A) CD31-, (B) CD44+, (C) CD45-, and (D) CD90+.

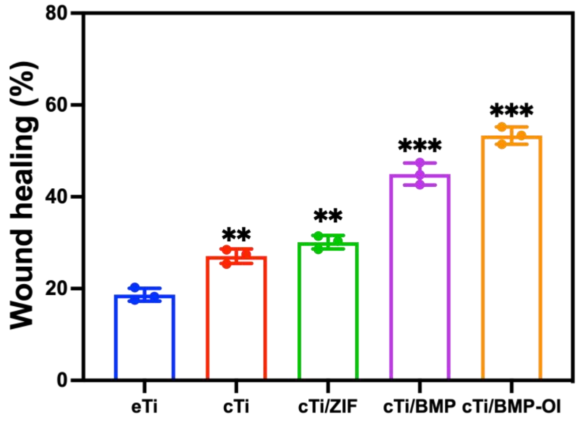

Figure S10. Evaluation of wound healing rates. (n = 3; data shown represent mean ± SD; **$p < 0.01$, ***$p < 0.001$).

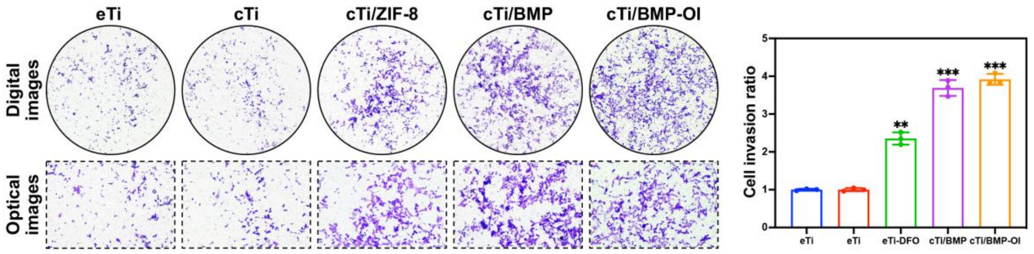

Figure S11. Impacts of eTi, cTi, cTi/ZIF-8, cTi/BMP and cTi/BMP-OI on BMSCs invasion and quantitative analysis of BMSCs stained with crystal violet. (n = 3; data shown represent mean ± SD; **p < 0.01, ***p < 0.001).

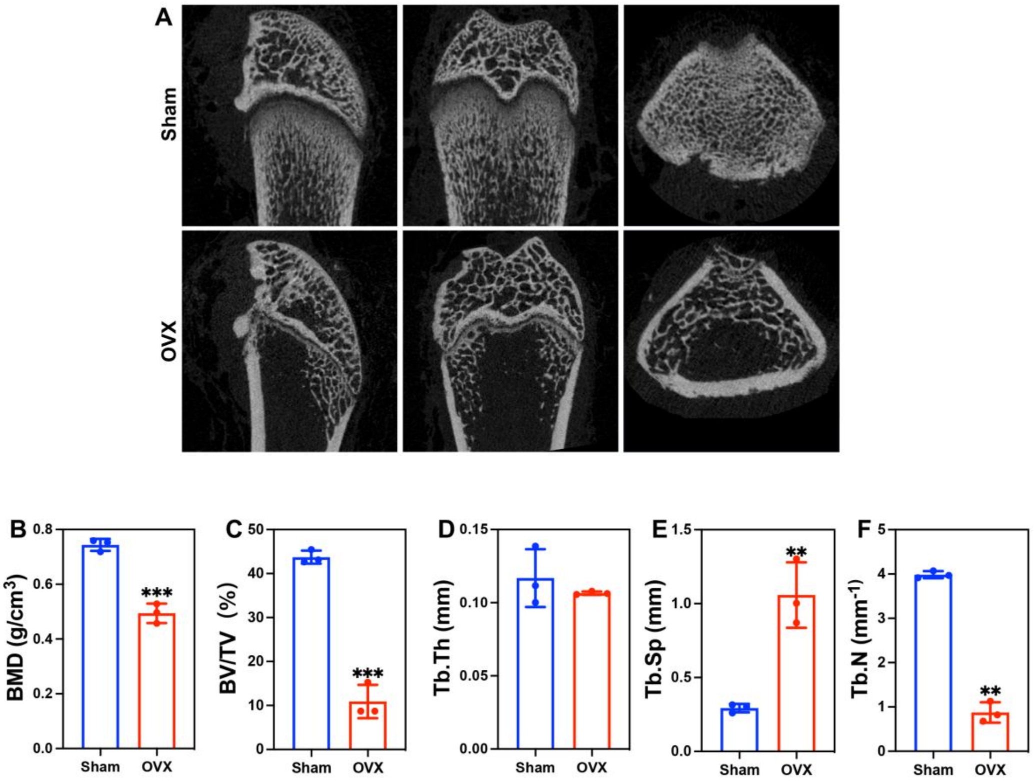

Figure S12. Validation of a rat model of osteoporosis. (A) Micro-CT 3D reconstruction between Sham and OVX. (B-F) Quantitative analysis of bone volume fraction (BMD, BV/TV, Tb.Th, Tb.Sp, and Tb.N). (n = 3; data shown represent mean ± SD; **p < 0.01, ***p < 0.001).

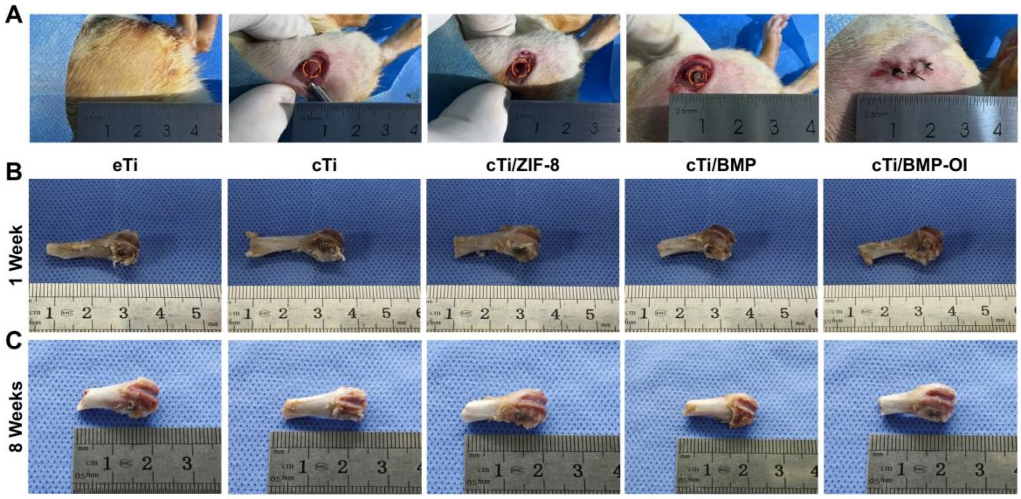

Figure S13. (A) Surgical steps for creating cylindrical bone defects and implanting scaffolds in the distal femur of rats. (B) and (C) Rat femur specimen with scaffold.

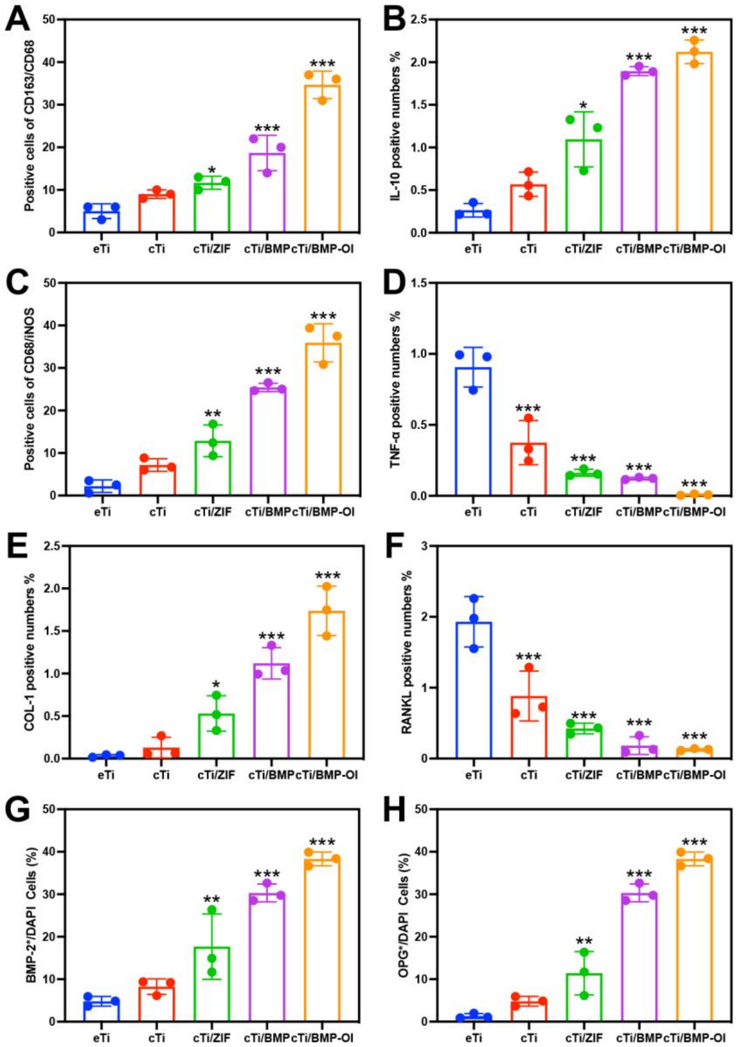

Figure S14. (A) and (C) Ratio of CD163⁺/CD68⁺ and CD68⁺/iNOS⁺ positive cells in tissue sections. (B), (D), and (E-F) Immunohistochemically stained images present the results ofthe statistical analysis ofprotein expression. (G) and (H) Ratio of BMP-2⁺ and OPG⁺ positive cells in tissue sections. (n = 3 biologically independent samples per group. Data are presented as mean ± SD. Statistical analysis was performed using one-way ANOVA followed by Tukey's post hoc test for multiple comparisons. $^{*}p < 0.05$, $^{**}p < 0.01$, $^{*}p < 0.001$.)

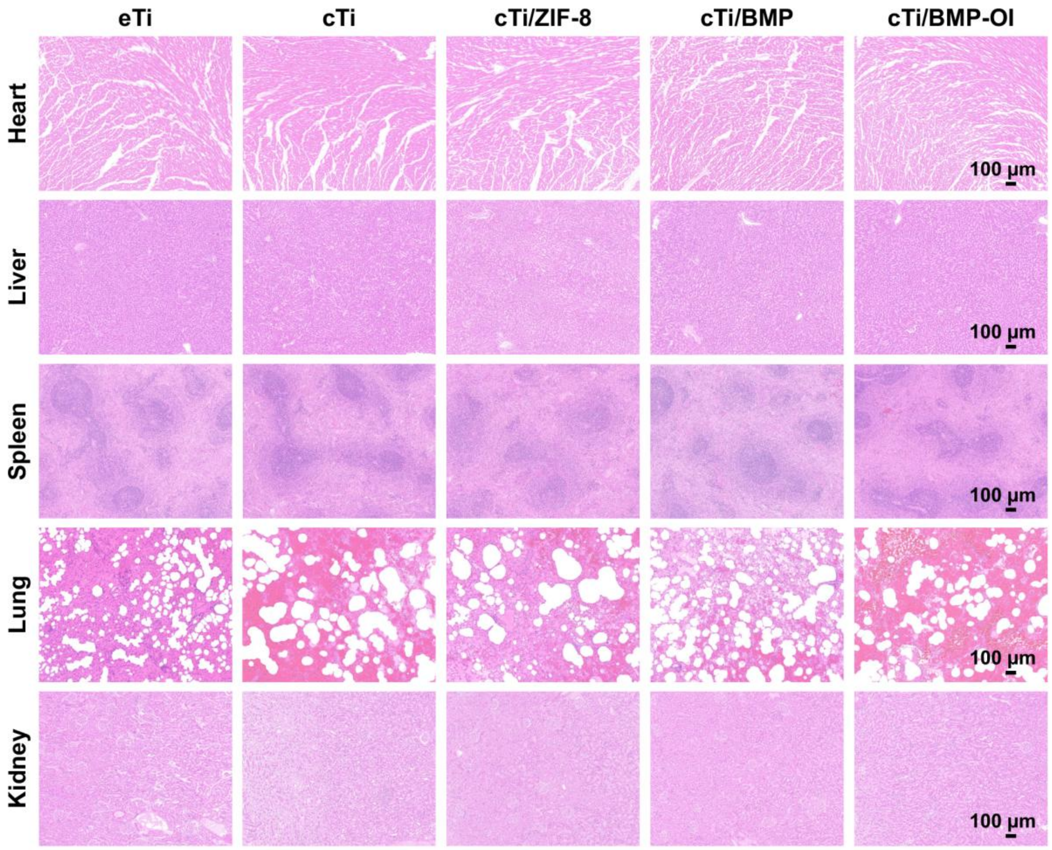

Figure S15. HE staining of different groups of major viscera at 8 weeks of implantation: heart, liver, spleen, lungs and kidneys.

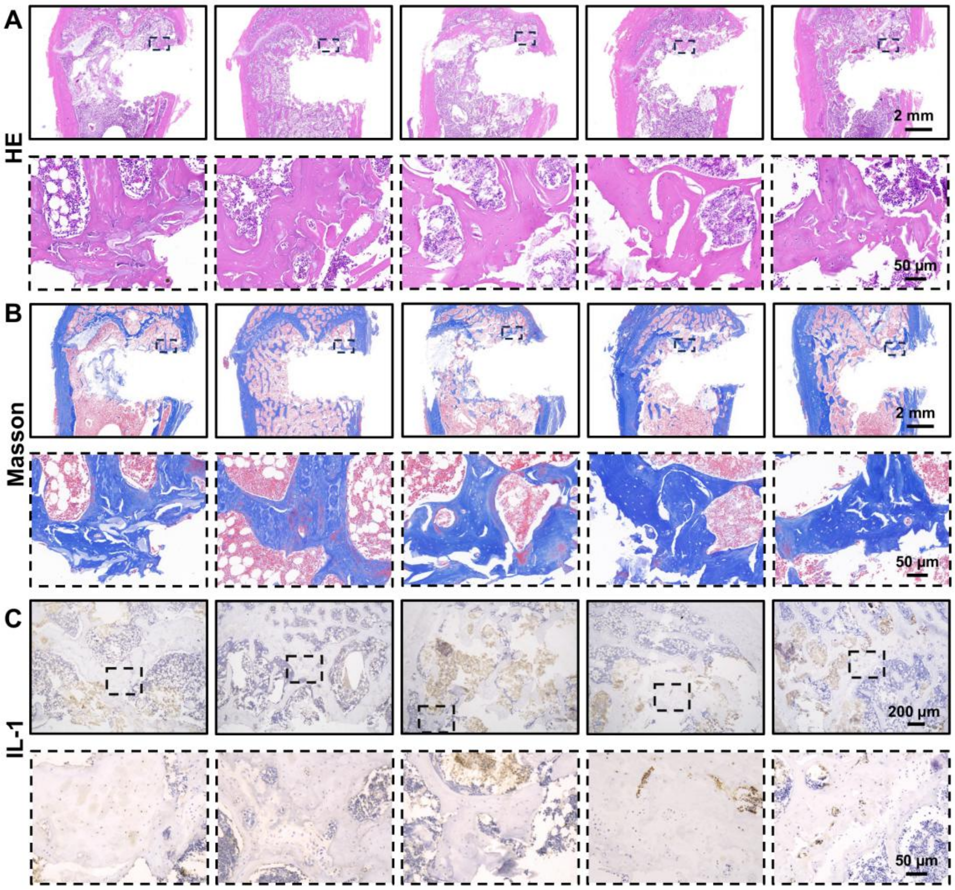

Figure S16. (A) HE and (B) Masson staining of the bone defect 8 weeks postimplantation. (C) Images of immunohistochemical staining of IL-1 in the peri-implant tissue (8 weeks).

Table S1 Primers used in RT-qPCR.

Gene	Forward primer	Reverse primer
GAPDH	CCTCTATGACAACACAGT	AGCCACCAATCCACACAG
ALP	CCGTGGCAACTCCATCTTT	CCTGGTAGTTGTTGTGAGCGTA
Col- I	CTGGTACATCAGCCCAAAC	GAACCTTCGCTTCCATACTC
Runx2	ATCATTCAGTGACACCACCA	GTAGGGGCTAAAGGCAAAAG
OPN	GAACATGAAATGCTTCTTTCTCAG	TCCATGAAGCCACAAACTAAACTA
NFATc1	GGAGCGGAGAAACTTTGCG	GTGACACTAGGGGACACATAACT
Cathepsin K	GAAGAAGACTCACCAGAAGCAG	TCCAGGTTATGGGCAGAGATT
GAPDH	AGGTCGGTGTGAACGGATTTG	TGTAGACCATGTAGTTGAGGTCA
